# Developing Explicit Base Stacking Potentials for the IsRNA2+ Coarse-Grained RNA Force Field Using Iterative Reweighting

**DOI:** 10.1021/acs.jctc.6c01116

**Published:** 2026-09-02

**Authors:** Wenfei Li, Shi-Jie Chen

**Affiliations:** † Department of Physics and Astronomy, 14716 University of Missouri-Columbia, Columbia, Missouri 65211-7010, United States; ‡ Department of Biochemistry, MU Institute of Data Science and Informatics, University of Missouri-Columbia, Columbia, Missouri 65211-7010, United States

## Abstract

Accurate prediction of RNA tertiary structures is essential for understanding their diverse biological functions. While machine learning methods have gained popularity, physics-based coarse-grained force fields remain an important tool for elucidating the structure and dynamics of RNA molecules. In this paper, we introduce the iterative reweighting algorithm as a flexible framework for optimization of force field parameters. We apply this framework to develop the IsRNA2+ force field, where the IsRNA force field is augmented by an explicit base-stacking potential. The performance of the IsRNA2+ force field is validated through structure prediction tasks on a benchmark set of 46 “simple” and 20 “difficult” RNAs. Compared to the original IsRNA2 force field, the current IsRNA2+ force field leads to improved accuracy in structure prediction. A detailed analysis of several cases with large improvements highlighted the importance of a balanced treatment of local energy components in determining the overall tertiary structures of RNA molecules. We also envision the potential of combining the machine learning approach with traditional force fields to further improve the understanding of RNA structures and dynamics.

## Graphical abstract

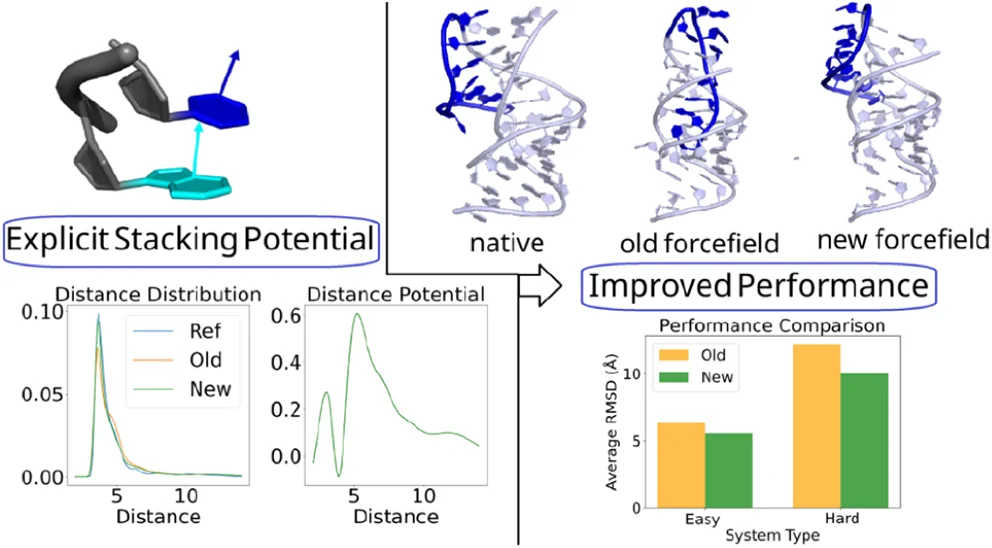

## Introduction

RNA molecules are important biomolecules with diverse cellular roles, such as regulating gene expression, facilitating protein synthesis, and catalyzing biochemical reactions. The functions of RNA molecules are closely related to their tertiary structures, and extensive efforts have been made in the computational prediction of RNA structures.^1–7^ These methods for RNA structure prediction can be classified into three major types: physics-based,^8–15^ knowledge-based,^16–25^ and machine learning-based.^26–31^ Physics-based methods typically involve the development of molecular force fields for the evaluation of RNA structures. In many cases, coarse-grained force fields are adopted for computational efficiency. We note that while machine learning-based methods are enjoying increasing popularity in recent years, physics-based methods remain a valuable direction of exploration, as an accurate coarse-grained force field will be beneficial not only for structure determination but also for studying the folding kinetics of RNA molecules.^32–37^

In the training of force field parameters, inverse Boltzmann methods^38,39^ are commonly used. The distributions of individual degrees of freedom are inverted independently to extract potentials according to the Boltzmann distribution, and the collective effects of different degrees of freedom are included by iteratively performing parameter optimization and molecular dynamics simulations. For example, in the development of the IsRNA force fields,^12,40,41^ parameters are gradually added at different iterations. The correlations between different degrees of freedom are absorbed into individual potential terms through the iteration process, and as a result, the values of these terms are dependent on the order of addition, although their sum (namely, the total potential energy) may remain independent of the order.

Here, we introduce the iterative reweighting algorithm^42,43^ as another versatile method for training force field parameters. In the reweighting algorithm, the Boltzmann factor is applied to each frame in a simulated trajectory, and the factor takes into account the contributions of all parameters to be optimized. In this way, the collective effects of different degrees of freedom are implicitly taken into account during each training step. We applied the method to train an explicit stacking potential as an augmentation to the original IsRNA2 force field. The performance of the updated force field is validated by performing structure prediction tasks on RNA molecules with different complexities, and the results indicate that a balanced representation of local energy components can have a large influence on the overall tertiary structures of the predicted models.

## Methods

### The Stacking Potential

Stacking between nucleobases is not only important for the stability of helical structures but also participates in the formation of many tertiary structural motifs of the RNA molecules.^44–46^ Base–base stacking is intrinsically a many-body interaction, characterized by interparticle distances and angles between the bases. In the IsRNA model,^12^ each residue is represented by five coarse-grained beads. Two beads, located at P and C4′ atoms, respectively, are used to trace the RNA backbone. Three beads are used to represent the base moieties, allowing IsRNA2 to treat noncanonical base pairing interactions. Base-stacking interactions in the original IsRNA models^12,41^ are accounted for by the collective contributions of pairwise potentials between beads belonging to the stacked bases. The same pairwise parameters are also used to characterize other interactions, such as tertiary interactions between distant bases. However, pairwise potentials pose a challenge when describing many-body interactions. We note that many coarse-grained RNA potentials treat stacking potential in a distinct manner. For example, in the Martini force fields of RNA and DNA molecules^47,48^ due to the thin, planar geometry of the nucleobases, special bead types were adopted for representing base-stacking interactions. In the IsRNA force fields,^12,41^ the general additive pairwise potentials may fall short in giving a full account of base-stacking interactions. As a result, the purpose of this work is to incorporate an explicit base-stacking potential into the IsRNA2 force field. It will serve as an additional correction term, not a replacement, of the stacking effect already captured by the original pairwise potentials.

As secondary structure constraints are applied in the training of the IsRNA2 force field, the helical geometries are well preserved in simulations, and statistical analysis of all stacked base pairs would be dominated by the helical regions. Therefore, to remove the helix bias, we chose to focus on stacking interactions between neighboring bases in loop regions. As the range of loop residues is obtained from the supplied secondary structure input, we emphasize that the performance of the IsRNA2+ force field will depend on the quality of the secondary structure.

One algorithm for characterizing base stacking is to consider three geometrical quantities, namely the distance between centers of the stacked bases, the angle between the normal vectors of the two base planes, and the angle between the normal vector of one base and the vector connecting the centers of the stacked bases.^49^ In this way, base stacking is identified as two bases in close distance, with parallel geometry and a substantial horizontal overlap area. The adoption of a three-bead representation in IsRNA2 allows us to define the centers and normal vectors of the nucleobases, and we choose the following form for the stacking potential between an adjacent pair of nucleobases with indices *I*,*J*:

1 $$V_{t,IJ}^{stack}=\left[V_{t}^{dist}\left(r_{IJ}\right)+V_{t}^{angle}\left(\theta_{IJ}\right)\right]\cdot w\left({\mathrm{r̂}}_{IJ},{\mathrm{n̂}}_{I},{\mathrm{n̂}}_{J}\right)$$

where *t* is a type index that designates three different types of stacked bases, depending on the identities of residues *I* and *J*: either both are purines, or one purine and one pyrimidine, or both are pyrimidines. As shown in Figure 1, *r*_*IJ*_ is the distance between the geometrical centers of the three coarse-grained beads on the two bases (hereby referred to as the stacking distance) and *r*_*ÎJ*_ is the corresponding normal vector; *n̂*_*I*_ and *n̂*_*J*_ are the normal vectors of the planes of the two bases, respectively, and θ_*IJ*_ is the angle between these two vectors (hereby referred to as the stacking angle). *V*_dist_ and *V*_angle_ are the distance and angle potentials to be trained, and the detailed training procedure will be discussed in later sections. A weight factor is adopted from the HiRE-RNA model,^14^ which encourages bases to stack on top of each other, rather than displaced. This term is fixed for the stacking potential and not updated through parameter training:

2 $$w\left({\mathrm{r̂}}_{IJ},{\mathrm{n̂}}_{I},{\mathrm{n̂}}_{J}\right)=\left(1-|{\mathrm{r̂}}_{IJ}\times {\mathrm{n̂}}_{I}{|}^{4}\right)\cdot \left(1-|{\mathrm{r̂}}_{IJ}\times {\mathrm{n̂}}_{J}{|}^{4}\right)$$

**Figure 1:**
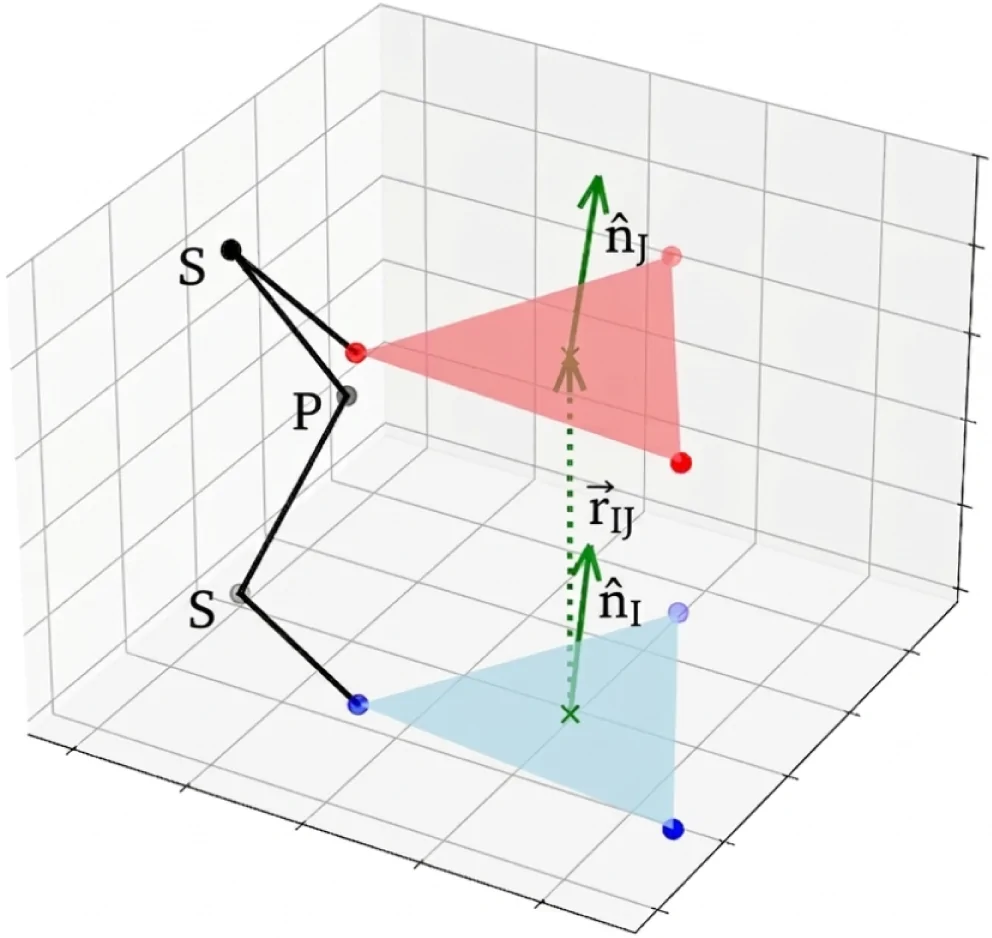
Schematic illustration of relevant geometrical quantities in the stacking potential. The backbone beads are shown in black, with S and P beads placed at C4′ and P atoms, respectively. The two adjacent bases are shown in blue and red. Each base is represented by three coarse-grained beads.^12^ The two solid arrows labeled *n̂_I_* and *n̂_J_* are the normal vectors of the two base planes, and the dotted arrow *r⃗_IJ_* is the displacement vector between the centers of the two bases.

The form of the weight factor is well-suited for the three-bead IsRNA2 model as all terms are readily available, and, in practice, simulations performed without it occasionally resulted in nonphysical geometries where bases were parallel but horizontally displaced (coplanar). By incorporating this weight factor, we effectively eliminated such erroneous behavior, ensuring correct vertical stacking geometries.

We will henceforth refer to the new force field as the IsRNA2+ force field.

### The Training Algorithm

An algorithm based on iterative reweighting^42,43^ is adopted for the training of the stacking potentials. Consider two potentials: the original potential *V*_1_(*x⃗*) and the updated potential *V*_2_(*x⃗*). In the context of this work, the original potential is the IsRNA2 potential, and the updated potential is the current (modified) IsRNA2+ potential with the additional stacking term. We suppose an equilibrium trajectory of *V*_1_ is already obtained, with *N* frames: {*x⃗*_*i*_,*i* = 1,···,*N*}. The two potentials will then assign different potential energies to the frames in the trajectory, denoted by {*V*_1_(*x⃗*_*i*_)} and {*V*_2_(*x⃗*_*i*_)}. Then, under *V*_2_, each frame will be reweighted by the following Boltzmann factor: If the difference between the potentials Δ*V* = *V*_2_ – *V*_1_ can be treated as a small perturbation, in which case we can assume the trajectory under *V*_1_ also gives a fair sampling of the conformational space under *V*_2_, with each frame reweighted by the Boltzmann factor:

3 $$w_{i}^{\left(2\right)}=e^{-(V_{2}({\mathrm{x⃗}}_{i})-V_{1}({\mathrm{x⃗}}_{i}))/k_{B}T},i=1,2,...,N$$

The reweighted trajectory can be taken as an approximation of equilibrium trajectory for *V*_2_. If the above approximation holds, then for an observable *Ô*, the ensemble-averaged value under the original potential *V*_1_ is given by a simple average over all frames:

4 $${\mathrm{O̅}}^{\left(1\right)}=\frac{1}{N}\overset{N}{\underset{i=1}{\sum }}Ô\left({\mathrm{x⃗}}_{i}\right)$$

while the ensemble-averaged value under the new potential *V*_2_ can be approximated by a reweighted average of the same frames:

5 $${\mathrm{O̅}}^{\left(2\right)}=\frac{1}{\overset{N}{\underset{i=1}{\sum }}w_{i}^{\left(2\right)}}\overset{N}{\underset{i=1}{\sum }}Ô\left({\mathrm{x⃗}}_{i}\right)\cdot w_{i}^{\left(2\right)}$$

The reweighted average *O̅* ^(2)^ can then be compared with some reference value *O̅* ^(ref)^ that we deem as the “ground truth” to be reached, and their difference could serve as the loss function for optimizing parameters in the potential *V*_2_. In practice, such ground truth is typically obtained from statistical analysis of the experimental structures or from all-atom simulations.

Alternatively, if the reference distribution of *Ô* is known, a comparison of distributions is often of interest, as distributions contain more information than simple ensemble averages. For practical purposes, the distributions are discretized before they are compared. Suppose the range of values taken by the observable *Ô* is binned into a series of small intervals: {*o*_1_,*o*_2_,···,*o*_*j*_,*o*_*j*+1_, ...,*o*_*M*_}, the original probability distribution under potential *V*_1_ is thus given by the proportion of frames falling into each bin:

6 $$p_{j}^{\left(1\right)}=\frac{1}{N}\underset{i}{\sum }{|}_{o_{j}<Ô\left(\overset{⃗}{x_{i}\;}\right)<o_{j+1}}1,j=1,2,...,M-1$$

while the reweighted probability distribution is approximated by a summation over the Boltzmann weights:

7 $$p_{j}^{\left(2\right)}=\frac{1}{\underset{i}{\sum }w_{i}^{\left(2\right)}}\underset{i}{\sum }{|}_{o_{j}<Ô\left(\overset{⃗}{x_{i}\;}\right)<o_{j+1}}w_{i}^{\left(2\right)},j=1,2,...,M-1$$

The reweighted probability can then be compared with some reference probabilities $\left\{p_{j}^{\left(\mathrm{ref}\right)},j=1,2,...,M-1\right\}$ . The loss function could be a straightforward summation over the absolute errors $\underset{j}{\sum }\left|p_{j}^{\left(2\right)}-p_{j}^{\left(\mathrm{ref}\right)}\right|$ , or other measures such as the widely used Kullback–Leibler divergence:^50^

8 $$D_{KL}\left(p^{\left(ref\right)}||p^{\left(2\right)}\right)=\underset{j}{\sum }p_{j}^{\left(ref\right)}\log\frac{p_{j}^{\left(ref\right)}}{p_{j}^{\left(2\right)}}$$

or the symmetric Jensen-Shannon divergence (JSD):^51^

9 $$D_{JS}\left(p^{\left(ref\right)},p^{\left(2\right)}\right)=\frac{1}{2}\left(D_{KL}\left(p^{\left(ref\right)}||p^{\left(2\right)}\right)+D_{KL}\left(p^{\left(2\right)}||p^{\left(ref\right)}\right)\right]$$

In practice, however, it is commonly the case that the trajectory under *V*_1_ cannot give a fair sampling of the conformational space under *V*_2_, either because the difference Δ*V* is large or that the conformational space is very complicated, such as in the case of large RNA molecules. To address this problem, an iterative procedure can be adopted.^43^ The procedure is similar to the iterative Boltzmann algorithm, where parameter optimizations and molecular dynamics simulations are iterated until convergence. A schematic illustration of the iterative reweighting framework is given in Figure 2.

**Figure 2:**
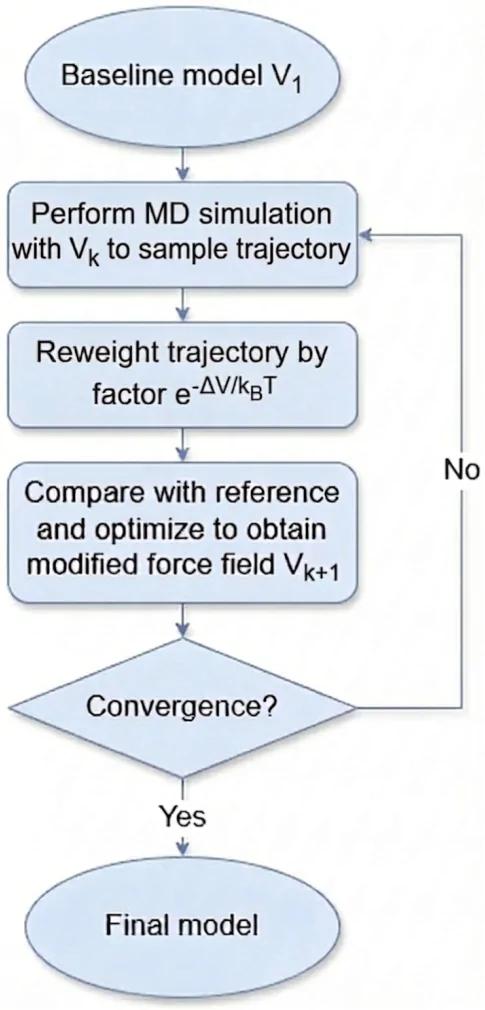
Schematic illustration of the iterative reweighting procedure.

Additionally, for the sake of simplicity, we presented the iterative reweighting method where only one observable *Ô* is considered. The method is easily extended to the scenario where the joint distributions of multiple observables {*Ô*_1_,*Ô*_2_,···} are of interest. The reweighted probabilities are again obtained as summations over Boltzmann weights, only this time for frames falling into multidimensional bins.

In addition, as mentioned in the Introduction section, we would like to emphasize again the difference between the reweighting algorithm and Boltzmann inversion. In Boltzmann inversion, the distributions of individual degrees of freedom are inverted independently to obtain corresponding potentials. The correlations between different degrees of freedom are not taken into account simultaneously in each iteration, but rather incorporated implicitly through the iteration process. This is more evident in the modified iterative Boltzmann inversion method adopted in the training of IsRNA force fields,^40^ where the correlation effects are absorbed into different individual terms depending on the order of iterations. On the other hand, in the current reweighting algorithm, energies of frames {*V*_*k*_(*x⃗*_*i*_)} (*k* = 1, 2) contain contributions from all force field parameters being optimized, so the correlations between parameters are naturally included in each iteration of the training process.

Putting it mathematically, consider two degrees of freedom, *x*_1_ and *x*_2_, with a joint distribution *p*_1_(*x*_1_,*x*_2_) under the initial potential *V*_1_ and a reference joint distribution *p*_*ref*_(*x*_1_,*x*_2_). Then, with the original Boltzmann inversion method,^38,39^ potentials of *x*_1_ and *x*_2_ are corrected independently:

10 $$\Delta V=\Delta V\left(x_{1}\right)+\Delta V\left(x_{2}\right)=k_{B}T\ln\frac{p_{1}\left(x_{1}\right)}{p_{ref}\left(x_{1}\right)}+k_{B}T\ln\frac{p_{1}\left(x_{2}\right)}{p_{ref}\left(x_{2}\right)}$$

In this case, the distribution of *x*_1_ under the corrected potential becomes

$$\begin{array}{ll} p_{2}\left(x_{1}\right) & =\int p_{2}\left(x_{1},x_{2}\right)dx_{2}=\int p_{1}\left(x_{1},x_{2}\right)e^{-\Delta V}dx_{2}\\ & =\int p_{1}\left(x_{1}|x_{2}\right)p_{1}\left(x_{2}\right)\frac{p_{ref}\left(x_{1}\right)}{p_{1}\left(x_{1}\right)}\frac{p_{ref}\left(x_{2}\right)}{p_{1}\left(x_{2}\right)}dx_{2}\\ & =p_{ref}\left(x_{1}\right)\int \frac{p_{1}\left(x_{1}|x_{2}\right)}{p_{1}\left(x_{1}\right)}p_{ref}\left(x_{2}\right)dx_{2} \end{array}$$

In the case where *x*_1_ and *x*_2_ are independent of each other, the integral evaluates to 1, and the distribution *p*_2_(*x*_1_) matches its reference. But if there is correlation between *x*_1_ and *x*_2_, *p*_2_(*x*_1_) does not match its reference. The problem lies in calculating Δ*V*(*x*_1_) and Δ*V*(*x*_2_) individually, where the correlation effect will be present in both terms and is therefore double-counted in the final Δ*V*. In the original Boltzmann inversion method, this double-counting problem is solved by incorporating an iterative framework, where MD simulation and parameter optimization are carried out iteratively. The overcompensated correlation term will affect the results of the MD simulation and is therefore corrected in the next parameter optimization step, where the distributions from the new simulation are compared with reference.^38^

Similarly, in the modified iterative Boltzmann inversion of IsRNA,^12,40,41^ this problem is solved by adding *x*_1_ and *x*_2_ separately in two iterations. In the first iteration, the correlation is absorbed into the potential for *x*_1_. The correlation effect will manifest in the following MD simulation; thus, in the second iteration, the added potential for *x*_2_ includes only the contribution from *x*_2_, excluding the correlation.

On the other hand, the trajectory reweighting algorithm incorporates the correlation effect within each iteration by working directly with the total potential correction Δ*V*(*x*_1_,*x*_2_). The form of Δ*V* can still be additive, namely Δ*V* = Δ*V*(*x*_1_)+Δ*V*(*x*_2_), but instead of inverting Boltzmann weights to calculate individual potential terms, we now search for a correction Δ*V* that directly gives $\left\langle e^{-\Delta V/k_{B}T}\right\rangle \approx p_{ref}\left(x_{1}\right)$ , where ⟨···⟩ denotes the distribution of *x*_1_ across the MD frames generated under the initial potential *V*_1_ (see eq 9). Therefore, we propose that the iterative reweighting method offers a general and highly flexible approach for the optimization of force field parameters.

Additionally, we note that the loss function we used for comparing the reweighted and reference distributions is different from the one used in other approaches such as ODEM,^52^ where the binned distributions are compared using a maximum-likelihood estimator by assuming that fractions in bins follow independent normal distributions: $\underset{i}{\prod }N\left(f_{i},\Delta f_{i}\right)$ where *f*_*i*_ = *n*_*i*_ /*n*_*total*_ is the fraction of observed values falling in bin *i*, $\Delta f_{i}=\alpha \sqrt{n_{i}}/n_{total}$ with a manually selected coefficient α to measure the uncertainty of the fraction, and N is the density of the normal distribution. In the ODEM work, α is chosen to be 1000. In practice, it is found that the choice of this coefficient has a significant effect on the optimized potentials, and it is difficult to decide an optimal value. Therefore, we adopted the symmetric KL divergence here due to its computational stability as well as compatibility with the stochastic gradient descent algorithm we used for parameter optimization.

### Training Set and Training Procedure

In this work, the base model *V*_1_ is the IsRNA2 force field,^12^ and the correction Δ*V* is the stacking potential, defined in eq 1. More specifically, the weight term *w* remains in its fixed analytical form, while *V*_*dist*_(*r*) and *V*_*angle*_(θ) are trained using the iterative reweighting method. Depending on the identities of the participating bases, three individual sets of distance and angle potentials are obtained: Purine–Purine, Purine–Pyrimidine, and Pyrimidine–Pyrimidine. In the training procedure, we will aim at matching the joint distributions of stacking distance *r* and angle θ obtained from IsRNA2+ simulations with the reference.

In the training of the stacking potential, we are interested only in the stacking geometries in loops and not the more complicated geometries that may form in larger RNA molecules. Therefore, to allow for more efficient training, a group of 14 small RNA structures is selected as the training set. In the construction of the training set, we have particularly focused on including structures with different loop lengths and types. The collection of structures has hairpin loops with lengths varying from 3 to 16, and internal loops with lengths ranging from 1 to 4. Furthermore, the purine fraction of the loops varies from 0.14 to 1.0, with an average of 60%. An analysis of the loop properties of the test set, shown in Supporting Information Figures S1 and S2, indicates that most loops fall in the range of the test set. The PDB IDs and relevant information on the 14 structures are listed in Table 1. Reference distributions are generated using all-atom Molecular Dynamics (MD) simulations. Initial configurations are prepared with the tleap package.^53^ RNA molecules are modeled using the ff99+bsc0+chiOL3 force field^54,55^ and solvated in TIP3P water^56^ at 150 mM NaCl concentration. A distance of 15 Å is used between the solute and box edges. The systems are first subjected to energy minimization and then heated to 298 K and equilibrated at 298 K and 1 bar for 0.1 ns using the pmemd program.^57^ The output configurations are then converted to the Gromacs format with the ParmED package,^58^ and production runs are carried out using Gromacs^59^ with a 2 fs time step. Trajectory lengths vary from 100–400 ns for different systems, depending on the time required for relevant distributions to converge. Frames are collected every 10 ps to calculate the reference distributions of stacking distances and angles in the loop regions.

**Table 1:** Loop Lengths and Purine Fractions of the 14 RNA Systems Used in the Training Set^a^

PDB ID	Internal Loop Length (nt)	Hairpin Loop Length (nt)	Purine Fraction
157D	1,1,1,1	-	0.50
1ATO	-	7	0.14
1D0U	1	4	0.60
1F85	-	4	0.50
1F9L	2,2	4	1.00
1FHK	-	8	0.50
1K5I	-	5	0.40
1KKA	-	5	0.80
1T28	-	16	0.63
2L8F	4,1,4,1	-	0.70
2LDL	1,1	7	0.56
2LX1	4,1,4,1	-	1.00
2NBY	1,1,1,1,2	3	0.56
402D	2,2	-	0.50

a If multiple loops are present, their lengths are separated by commas (″,″). A hyphen (″-″) indicates the absence of the corresponding loop type.

In each iteration, coarse-grained simulations are carried out with the OpenMM package^60^ at 300 K and 1 bar, with a 0.5 fs time step for 100 ns. Frames are collected every 2.5 ps to calculate the distributions of stacking distances and angles in the loop regions. Secondary structure constraints are applied in the simulations to maintain the stability of helical regions. As OpenMM supports tabulated numerical potentials on grids, we did not specify any functional form of the potentials *V* ^dist^(*r*) and *V* ^angle^(θ). Instead, the distance potential is defined on the range of [2,14) Å, while the angle potential is defined on the range of [−0.1,π+0.1) radians. A total of 100 equally spaced grid points are used to partition the ranges, and numerical values of *V* ^dist^(*r*) and *V* ^angle^(θ) on these grids are optimized through iterative reweighting.

Parameter optimizations are performed using the stochastic gradient descent algorithm from the PyTorch module.^61^ The learning rate is set to be 0.005, and the optimization runs for 5000 epochs in each iteration. Apart from the Jensen–Shannon divergence between reference and reweighted distributions, a regularization term based on the sum of squared second derivatives is employed to avoid irregularities in the numerical potential, giving a loss function of the form:

11 $$L=\overset{3}{\underset{t=1}{\sum }}\left\{D_{JS}\left(p^{t,\left(ref\right)},p^{t,\left(reweighted\right)}\right)+\lambda \left[\int \left|\frac{d^{2}}{dr^{2}}V_{t}^{dist}{|}^{2}dr+\int \right|\frac{d^{2}}{d\theta^{2}}V_{t}^{angle}{|}^{2}d\theta \right]\right\}$$

where the summation runs over all three stacking types (Purine–Purine, Purine–Pyrimidine, and Pyrimidine–Pyrimidine) and λ is a weighting factor set to be 0.5 in the training process.

To ensure a robust force field, training was carried out to avoid excessive numerical oscillations and maintain a stable optimization process. The selection of the regularization weight λ was based on the training results of the first iteration. The values of Jensen–Shannon divergence and the regularization term at the final epoch for different λ values are shown in Supporting Information Figure S3, along with the final potentials for λ = 0.1 and λ = 0.5 in Supporting Information Figure S4. Both 0.1 and 0.5 appeared as feasible choices from the loss values; however, a closer inspection of the potentials revealed unwanted oscillations in the middle-range region (6–11 Å) for the pyrimidine–pyrimidine distance potential. Consequently, λ = 0.5 was adopted.

The training losses across all iterations are shown in Supporting Information Figure S5. The learning rate of 0.005 was chosen as a conservative choice for the stochastic gradient descent optimizer, and it produced a stable, monotonically decreasing loss curve that gradually plateaus at epoch 5000. For example, in the first iteration, the Jensen–Shannon divergence reduced by 36% across the training process, while it only reduced by 0.5% at the last 100 epochs. We also tested learning rates of 0.001 and 0.01 for the first iteration and reported the loss curves as well as the final potentials in Supporting Information Figures S6 and S7, respectively. The Jensen–Shannon divergence curves show that a rate of 0.001 is too conservative, as the loss is far from a plateau after 5000 epochs. On the other hand, the potentials trained with rates of 0.005 and 0.01 share similar peak and valley positions. As we adopt an iterative reweighting procedure, it is not necessary to obtain the maximum potential depth in a single iteration. As a result, we retained 0.005, aiming for a more stable training process.

Altogether, three training iterations were performed, and the potentials of each iteration as well as the distributions of stacking distances and angles are reported in Supporting Information Figure S8. Although the potentials of iterations 3 and 2 still show a difference, it has a minimal effect on the distributions of the simulation results (the Jensen–Shannon divergence between angle and distance distributions from simulations using iteration 2 and iteration 3 potentials range from 0.007 to 0.028). Therefore, to avoid possible overfitting to the training set, we adopted the potentials of iteration 2 as the final stacking potentials.

Here, we report partial distributions and potentials of stacking distances and angles in Figure 3. In the panels containing partial distributions, “reference” distributions are obtained from all-atom simulations, “initial” distributions are obtained from simulations using the IsRNA2 force field,^12^ and “final” distributions are the reweighted distributions of iteration 2. From the partial distributions, it is clear that the IsRNA2 force field does not take full account of the base-stacking interactions. The peaks in the distributions of both stacking distance and angle often fall short of the reference, all-atom distributions. Although the final distributions do not exactly match the reference due to the regularization term in the loss function, the corrections are quite clear from the plots.

**Figure 3:**
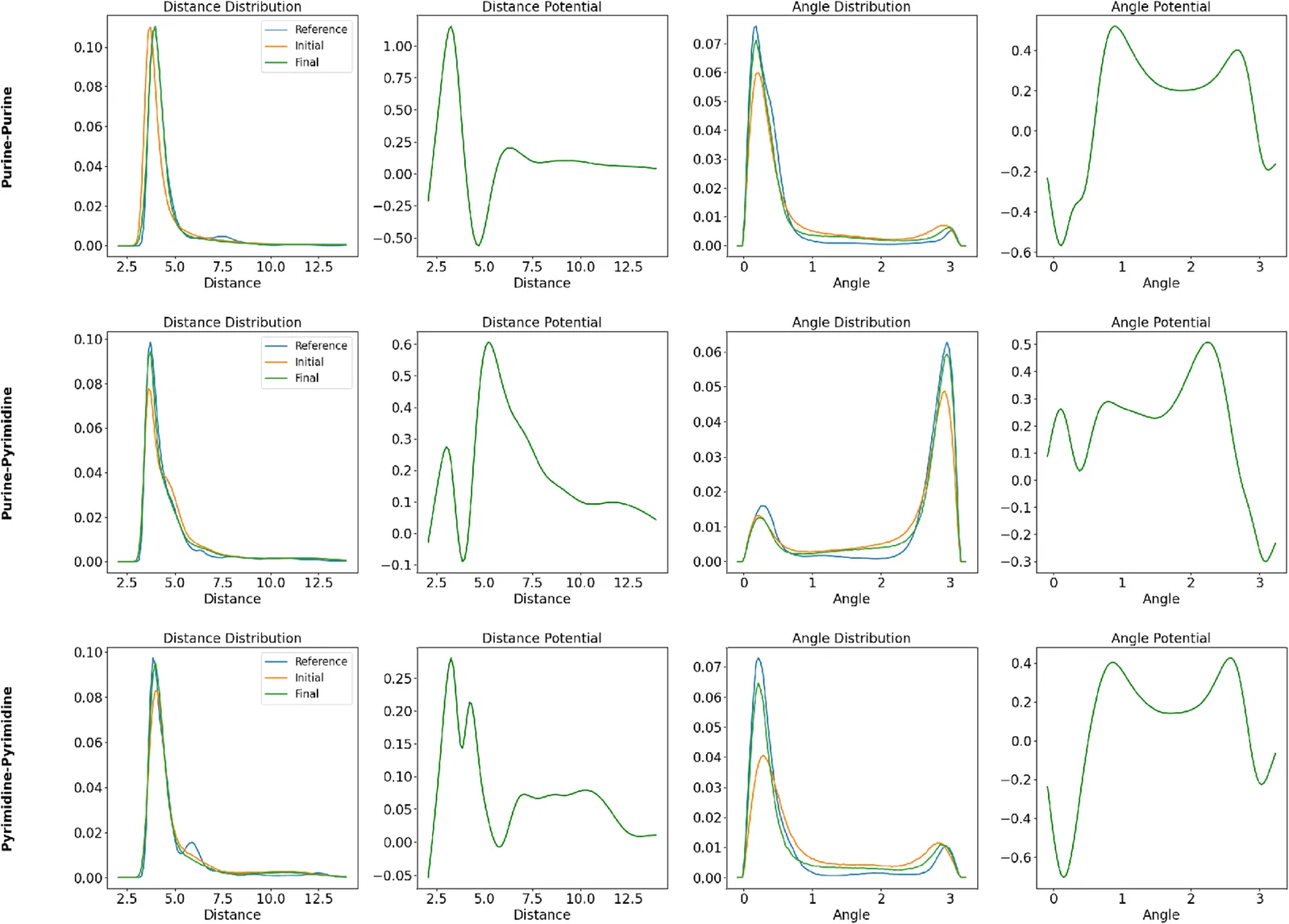
Figures showing training results for each base-stacking type (Purine–Purine, Purine–Pyrimidine, and Pyrimidine–Pyrimidine). For each type, the four panels from left to right contain the distributions of stacking distances, the distance potentials, the distributions of stacking angles, and the angle potentials, respectively. For the partial distributions, the reference, initial (simulated by the IsRNA2 potential), and final (reweighted distribution of the last training iteration) distributions are shown in blue, orange, and green. The units for distances, angles, and potential energies are angstroms, radians, and kcal/mol, respectively.

## Results and Discussion

### Overall Performance

To test the performance of the new stacking potential, we ran structure prediction with the IsRNA2+ force field. As this benchmark is focused on tertiary structure prediction, for models that require secondary structure as input, we used the native secondary structures extracted from PDB structures using the X3DNA-DSSR package.^62^ The test set can be divided into two groups: the first group consists of 46 simpler structures, including hairpins with and without internal loops, small pseudoknots, and up to four-way junctions; the second group are selected from structures classified as “difficult” in one recent benchmark work,^63^ consisting of more complicated structures that are made up of more than one junctions or pseudoknots. The structure prediction uses the same procedure as the original IsRNA force fields, where a replica-exchange molecular dynamics simulation is first carried out, followed by cluster analysis. The centroid structures of the largest 5 clusters are selected as candidates. Initial structures are assembled using the template-based method VfoldLA^20^ with sequence and secondary structure as input. Secondary structure constraints are applied to stabilize helical regions and to identify loop regions where the stacking potential will be applied. Additionally, we also ran structure prediction on the 14 systems in the training set. According to the classification criteria, they also belong to the “simple” type.

The results are compared with other methods: IsRNA2,^12^ SimRNA,^11^ FARFAR2,^8^ iFoldRNA,^15^ RNAJP,^13^ and AlphaFold3.^31^ With the exception of AlphaFold3, the same secondary structure constraints are applied for all prediction methods. The iFoldRNA runs are performed using the online server, while the other runs are performed using distributed packages on local machines. For AlphaFold3, 500 conformations are generated for each system with different random seeds; the structures are then clustered; and centroids of the top 5 clusters are selected. IsRNA2 and SimRNA both require initial tertiary structure input, and the same input structures as IsRNA2+ runs are used. For each method, default parameters provided by online servers or tutorial examples are adopted in the prediction runs to generate top 5 candidate structures. The best results among the 5 are reported in Table 2 (for simpler systems) and Table 3 (for “difficult” systems). The results of the 14 systems in the training set are provided in Supporting Information Table S1. Some results are missing for iFoldRNA and RNAJP because iFoldRNA does not support structures with multiple chains, RNAJP does not support structures with more than four-way junctions, and some systems cannot finish after multiple attempts.

**Table 2:** RMSD (in Å) of the Predicted Structures of 46 Simpler Systems Using Different Methods, Measured on Heavy Atoms^a^

	IsRNA2	IsRNA2+	SimRNA	iFoldRNA	FARFAR2	AlphaFold3	RNAJP
1A60	7.09	6.95	**6.06**	9.77	8.28	5.18	8.60
1E95	7.91	**3.68**	3.73	8.03	5.68	1.58	4.29
1FQZ	5.28	4.71	4.37	5.00	4.47	1.35	**2.79**
1KAJ	9.30	8.45	8.87	-	**6.66**	8.37	7.83
1KP7	5.29	5.26	4.94	**4.35**	6.16	2.23	6.71
1KPD	5.91	5.40	6.20	5.37	6.49	2.87	**4.99**
1L2X	3.51	3.22	**3.21**	4.46	4.18	2.15	3.46
1P5N	4.57	4.10	4.51	4.74	6.01	1.59	**3.37**
1Q9A	4.82	4.78	4.36	4.76	4.93	0.95	**1.53**
1RMN	9.27	9.24	**6.60**	-	9.32	6.55	8.67
1RNK	6.10	5.88	**5.75**	5.75	6.44	4.16	7.12
1U9S	15.30	**15.06**	24.86	-	28.59	20.44	23.55
1YG4	4.27	3.98	**3.74**	7.79	4.27	3.34	4.43
1YKQ	**3.08**	3.49	9.98	-	6.36	1.21	3.44
1YSV	1.87	1.93	**1.85**	2.37	4.25	0.88	2.76
299D	5.76	**5.63**	6.33	-	9.80	0.67	8.21
2A43	3.83	3.78	**3.56**	4.80	4.47	2.04	3.57
2AP5	**3.90**	4.08	4.17	6.90	5.38	2.08	5.31
2CKY	6.05	**5.65**	11.38	15.84	15.73	3.79	6.88
2G1W	4.97	**4.57**	4.83	5.08	5.85	1.91	4.98
2LDZ	3.70	**3.18**	3.85	4.05	4.22	2.31	3.55
2LU0	7.28	9.58	7.86	**5.47**	8.16	3.36	5.77
2MHI	14.80	12.04	14.26	14.83	**12.01**	7.77	12.10
2MTJ	10.56	**4.17**	7.17	-	9.92	5.76	10.61
2N6Q	3.18	3.15	**3.12**	5.35	5.21	2.67	3.48
2N8V	9.00	8.89	8.42	-	**8.32**	7.23	9.26
2NBX	14.60	15.79	**11.47**	16.94	13.42	11.02	14.41
2NC1	12.47	9.61	11.36	17.90	14.28	9.48	**7.83**
2OIU	12.10	9.37	11.94	15.11	9.58	11.23	**6.77**
2P89	7.71	6.77	6.04	-	8.39	1.53	**5.43**
2QUS	8.20	**5.39**	6.29	6.97	7.87	3.85	8.98
2QWY	**4.13**	4.17	5.57	9.21	6.52	2.57	6.45
2RP1	3.69	3.65	**3.64**	5.16	4.50	2.34	3.88
2TPK	5.75	**2.87**	3.20	4.63	5.55	2.26	6.02
3E5C	7.71	7.68	6.31	12.17	6.50	6.75	**5.85**
3LA5	9.80	5.33	7.11	6.57	**4.42**	1.64	6.77
3VRS	12.53	9.41	9.09	11.05	**8.38**	2.33	8.63
3ZD5	7.95	**6.89**	8.04	-	9.23	1.68	9.35
4JF2	13.64	**8.29**	10.99	12.10	9.63	2.39	12.89
4KZ2	**5.17**	5.59	8.82	-	7.19	0.88	5.72
4LVW	**3.73**	3.79	6.62	13.84	9.36	1.44	4.61
4OJI	3.09	**2.92**	3.81	-	7.72	1.36	3.83
4RGE	**3.26**	8.79	7.36	-	4.78	1.03	-
5KH8	9.91	9.11	**8.38**	11.13	9.80	2.63	9.04
8VM8	15.33	8.97	18.56	14.29	**7.06**	18.56	9.00
9IWF	11.57	12.52	11.05	7.70	7.53	5.13	**7.39**
**Average**	7.37	6.47	7.38	8.51	7.89	4.19	6.89

a Missing data points due to failed runs and restrictions of tested methods are marked by “-”. For each RNA molecule, the best result other than AlphaFold3 is shown in bold.

**Table 3:** RMSD (in Å) of the Predicted Structures of 20 Difficult Systems Using Different Methods, Measured on Heavy Atoms^a^

	IsRNA2	IsRNA2+	SimRNA	iFoldRNA	FARFAR2	AlphaFold3	RNAJP
2G9C	4.78	5.16	7.36	6.66	7.21	1.06	**4.60**
2XO1	4.16	**3.61**	8.23	7.72	8.62	1.15	4.94
3D0U	12.25	**10.27**	17.18	22.71	15.60	1.30	-
3DIG	21.59	18.94	17.58	25.21	**12.83**	1.33	-
3DS7	**4.88**	5.02	7.77	13.24	12.01	1.87	6.73
3F2Q	21.34	12.10	12.42	-	16.79	1.11	**8.46**
3GX2	9.73	**5.30**	10.72	13.25	14.70	0.87	15.00
3NPN	12.04	**6.30**	8.89	-	9.65	6.18	10.79
3RKF	7.08	**3.59**	5.07	5.22	8.99	1.30	4.45
3SKL	**3.65**	3.98	5.43	7.08	8.14	4.42	4.65
4GXY	25.44	19.03	21.99	-	25.14	6.13	**12.67**
4L81	12.11	14.49	13.57	**9.60**	19.70	2.83	14.02
4QK8	15.80	**8.83**	14.90	14.11	18.74	2.69	10.64
4QLM	14.86	13.24	**11.87**	-	15.35	5.58	13.43
4Y1M	11.71	**8.08**	13.93	14.83	19.50	2.07	10.44
5KPY	8.54	8.97	11.10	13.13	8.08	6.96	**8.06**
6N5K	15.75	16.38	**10.73**	17.82	15.92	2.91	17.01
6N5T	15.07	15.72	12.59	16.14	15.59	2.64	**12.15**
6VMY	14.20	12.36	**12.17**	23.50	21.82	21.70	14.94
7KVT	9.27	8.83	21.92	9.87	9.62	7.56	**8.47**
**Average**	12.21	10.01	12.27	13.76	14.20	4.08	10.08

a Missing data points due to failed runs and restrictions of tested methods are marked by “-”. For each RNA molecule, the best result other than AlphaFold3 is shown in bold.

To make a more comprehensive benchmark, we also tested on two models: oxRNA and HiRE-RNA, both of which have explicit stacking terms. The results are listed in Supporting Information Tables S2 and Table S3. For both methods, we used the same initial structure as the others. For oxRNA,^64^ we applied a secondary structure constraint and ran REMD at 6 different temperatures, equally spaced from 300 to 350 K, for 25000 time units. Clustering was performed using the default PCA procedure in oxDNA analysis tools. Cluster centroids from the top 5 largest clusters were selected as the predicted structures. For HiRE-RNA, REMD was carried out with the same setting as the example 1EOR in the original HiRE-RNA work.^65^ Cluster analysis was performed based on pairwise RMSD, and centroids from the top 5 largest clusters were selected as the predicted structures. Hire-RNA predictions were restricted to 18 systems in the “simple” test set with fewer than 40 nucleotides to ensure computational efficiency.

The average RMSDs of structures predicted by oxRNA and Hire-RNA on the 46(18) simple systems are 10.37 Å and 9.32 Å, respectively, while the average RMSD of structures predicted by oxRNA on the 20 hard systems is 14.65 Å. As highlighted in the Supporting Information, it is important to note that the two models were not originally designed for the specific task of RNA tertiary structure prediction. The oxRNA model was primarily developed to explore the structural, mechanical, and thermodynamic properties of RNA molecules and is capable of handling very large systems.^64^ And HiRE-RNA was designed to study RNA folding dynamics and kinetics.^14,65^ Also, unlike other methods in this benchmark, HiRE-RNA does not employ secondary structure constraints in its folding simulations, which frequently resulted in the unraveling of helical regions during REMD runs.

The first observation we make is that, because the test cases used here are all from the PDB and thus within the training set of AlphaFold3, as expected, AlphaFold3 outperforms all other methods in most cases. We also find that the accuracy of AlphaFold3 predictions does not seem to be overly affected by the complexities of the systems. Our finding is consistent with a previous benchmark work,^2^ where the performance of AlphaFold3 was shown to surpass all other tested methods, including ab initio, template, and other machine learning-based methods. It is important to note that AlphaFold3 still makes mistakes on this test set, sometimes rather large ones. The two very prominent cases are 1U9S and 6VMY, where the RMSDs of AlphaFold3 predicted structures exceed 20 Å. A closer look at the predicted structures indicates that the error of 1U9Soriginates from the flipping of the orientations of a pair of coaxially stacked helices (Figure 4a,b); in the predicted structure of 6VMY, the major difference lies in the orientation of the helix at the 3′-end (Figure 4c,d).

**Figure 4:**
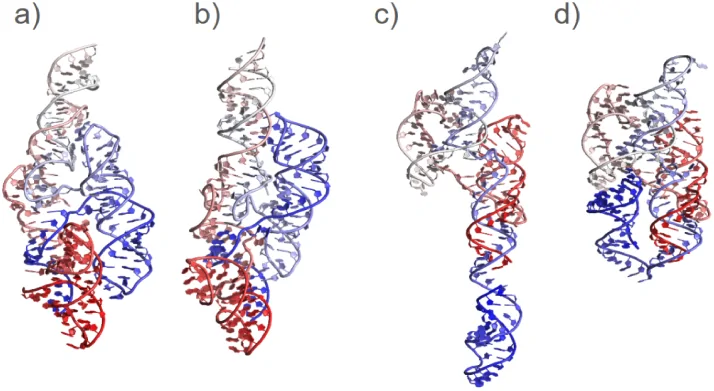
(a) Native and (b) AlphaFold3-predicted structures of RNA 1U9S. (c) Native and (d) AlphaFold3-predicted structures of RNA 6VMY.

Furthermore, while machine learning methods have led to great advances in the structure prediction of biomolecules, coarse-grained potentials remain important for the ability to elucidate the dynamics of biological pathways. Furthermore, by building physical models of biomolecules, we can use physical force fields to study the interactions with various environmental factors. Ultimately, the two methods are not mutually exclusive to each other, and it will be discussed later that there is the possibility of combining the strengths of both approaches.

Apart from AlphaFold3, it is clear that the inclusion of stacking potential improves the accuracy of predicted structures of the IsRNA2 force field. For the 46 simpler systems, the average improvement is less prominent, where average RMSD reduces by 0.90 Å. This is partly because both methods are able to give fairly accurate predictions for these simpler and smaller systems. But we still observe cases where the stacking potential about halves the RMSDs, such as for the iconic H-type pseudoknots 1E95 and 2TPK and the multiway junctions 2MTJ, 8VM8, and 3LA5 The improvements over more complicated systems are larger, where average RMSD reduces by 2.20 Å, and significant reductions of RMSDs are observed for 3F2Q, 3GX2, 4QK8, 3NPN and 3RKF.

Furthermore, *t* tests and Wilcoxon signed-rank tests were carried out for the two sets of systems. Both tests indicate that the improvement of the IsRNA2+ force field compared to IsRNA2 is statistically significant. The results are listed in Table 4.

**Table 4:** Statistical Significance Analysis Comparing the Prediction Accuracy (Measured in RMSD of Heavy Atoms) of IsRNA2 and IsRNA2+ Force Fields

Test Set	Test Type	Statistic	*p*-value	Significance
Simple Systems (*n* = 46)	Paired *t* test	*t* = 2.85	0.0065	Highly Significant (*p* < 0.01)
	Wilcoxon	*W* = 227.0	6.14 × 10^–4^	Highly Significant (*p* < 0.001)
Difficult Systems (*n* = 20)	Paired *t* test	*t* = 3.20	0.0047	Highly Significant (*p* < 0.01)
	Wilcoxon	*W* = 37.0	0.0094	Significant (*p* < 0.01)

In addition, while we have included a regularization term and remained conservative in the selection of training parameters, the rather small training set still raises questions about potential overfitting problem. To test the generalizability of the trained stacking potential, we also examined systems with at least one internal loop of length ≥5, as it exceeds the maximum length of internal loops in the training set. For simple systems, that gives 29 systems with an improvement of 1.16 Å, and for difficult systems, that gives 18 systems with an improvement of 2.04 Å. This performance indicates that the stacking potential can be generalized to loops with lengths exceeding those in the training set. One possible reason is that while the overall loop geometries may vary, the local physical interactions between adjacent bases remain relatively consistent.

### Visualization of Selected Cases

In this work, the stacking potentials are applied only to adjacent residues in loop regions. We classify this type of interaction as “local” in sequence space, in contrast to interactions involving residue pairs that are distant along the sequence but close in space. It is interesting how a “local” stacking interaction could lead to an overall conformational change in the predicted structures, as seen in many cases reported above. Therefore, in this section, we investigate a few cases where the stacking potential leads to large improvements compared to the original force field and discuss possible reasons for such improvements.

The first example is the H-type pseudoknot 1E95; see Figure 5. With secondary structure imposed, most of the structural flexibility stems from the orientation of loop L2 (residues 20–28, colored green in the figure). In the structure predicted by the original IsRNA2 force field (Figure 5b), the loop is attracted to the helices and bent toward the “interior” of the RNA molecule, leading to a very crowded structure. Steric hindrance in the middle region of the structure causes several disruptions in the stacking in loop L2. On the other hand, the L2 loop predicted by IsRNA2+ potential, shown in Figure 5c is close to that of a standard single-stranded RNA, where pairs of consecutive bases stack with each other. This is similar to the native structure, where, with the exception of a single-residue bulge, all other residues are well-stacked, forming a continuous single-stranded loop. Therefore, we propose that the stacking potential outweighs the attraction of the pairwise potential and helps to restore the integrity of the loop region, leading to a structure with the correct orientation of loop L2.

**Figure 5:**
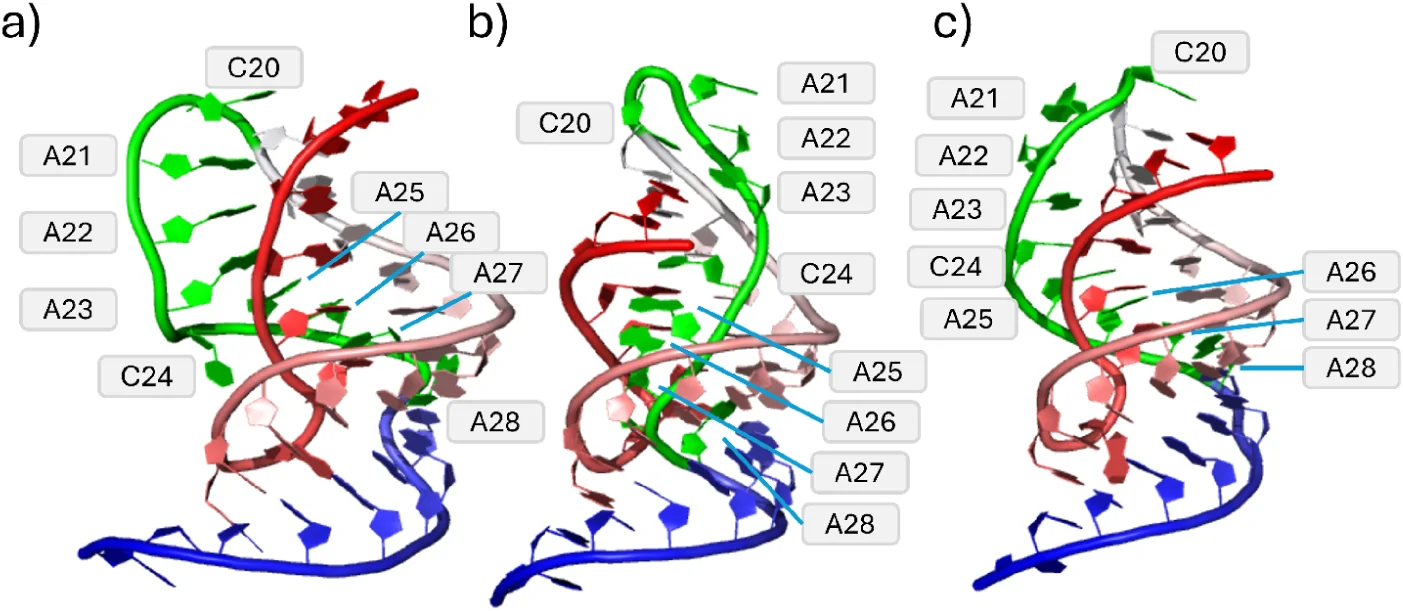
(a) Native structure; (b) structure predicted by IsRNA2;^12^ (c) structure predicted by IsRNA2+ of RNA 1E95, respectively. Residues in loop L2 are colored green and labeled. In the native structure (a), with the exception of the bulged-out C24, residues C20–U27 are all stacked in a consecutive manner. In the structure predicted by IsRNA2 (b), the stacking interactions between C20–A21, A21–A22, and A23–C24 are disrupted. In the structure predicted by IsRNA2+ (c), residues A21–A28 are all stacked consecutively.

The second example is the three-way junction 2MTJ; see Figure 6. The main difference between the IsRNA2-predicted structure (Figure 6b) and the native structure (Figure 6a) lies in the part of the loop containing residues 36 and 37, which are colored green in the figure. In the IsRNA2-predicted structure, this part of the loop is attracted toward the middle of the structure, and as a result, the stacking between residues 36 and 37 is disrupted. In the structure predicted by the IsRNA2+ potential (Figure 6c), the stacking geometry is restored along with the correct position of the loop.

**Figure 6:**
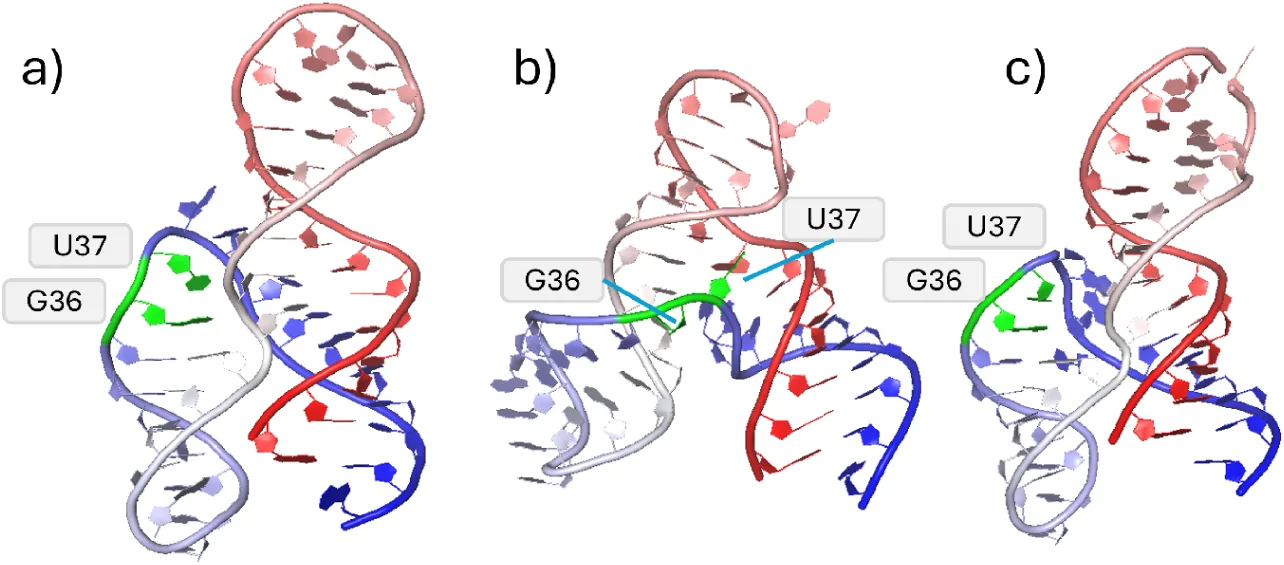
(a) Native structure; (b) structure predicted by IsRNA2; (c) structure predicted by IsRNA2+ of RNA 2MTJ, respectively. Residues 36 and 37 are colored green and labeled. The two residues are stacked in both the native structure (a) and the IsRNA2+-predicted structure (c), but disrupted in the IsRNA2-predicted structure (b).

The third example is the four-way junction 8VM8; see Figure 7. In the structure predicted by IsRNA2+ (Figure 7c), the helix–helix coaxial stacking patterns are consistent with the native structure (Figure 7a), although the orientations of the helices are slightly different. In contrast, the orientations and coaxial stacking patterns of helices in the IsRNA2-predicted structure (Figure 7b) are completely different from those of the native structure, with loops attracted toward each other, forming a very crowded middle region. This results in disruptions of stacking interactions between adjacent bases in various loops, such as residues 35 and 36, residues 74–76, and residues 83 and 84, which are highlighted using different colors in the figure. From the secondary structure plot (Figure 7d), it is clear that pairs 35 and 36 and 83 and 84 are important for the coaxial stacking patterns of the helices. Therefore, we propose that the stacking potential helps to preserve the coaxial stacking patterns around the junction region, leading to more-accurately predicted structures.

**Figure 7:**
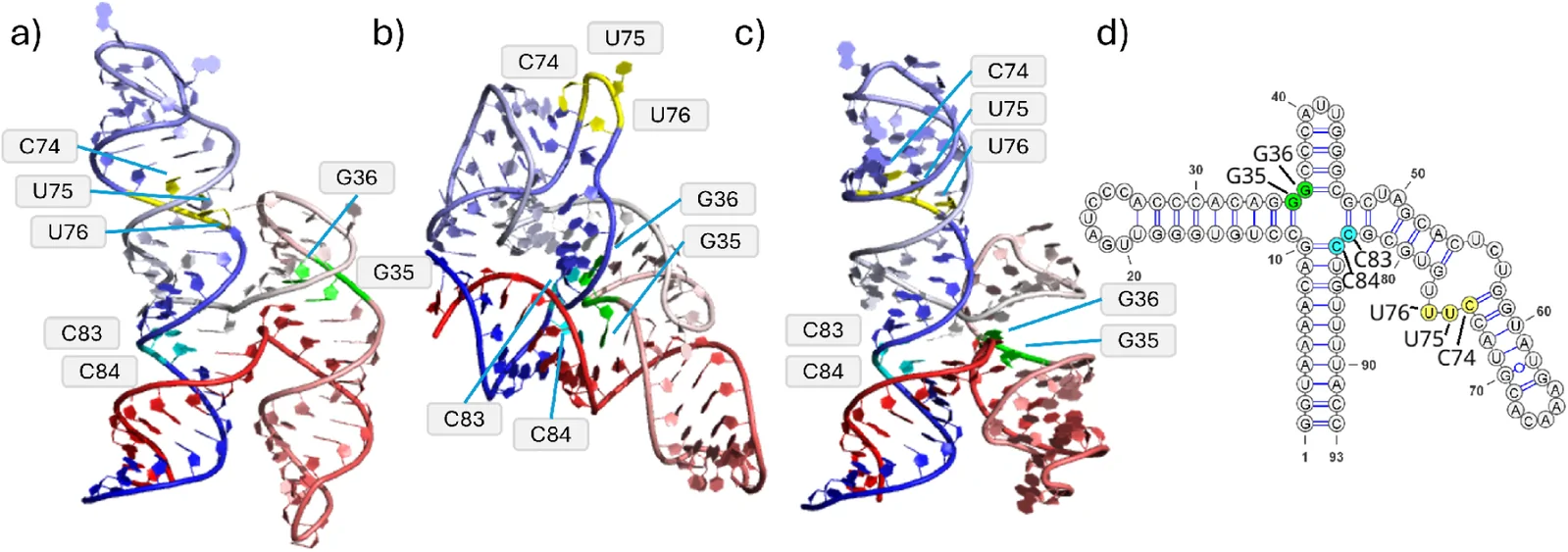
(a) Native structure; (b) structure predicted by IsRNA2; (c) structure predicted by IsRNA2+; (d) secondary structure of RNA 8VM8, respectively. Residues 35 and 36 are colored green, residues 74–76 are colored yellow, residues 83 and 84 are colored cyan and labeled. The residues are stacked in both the native structure (a) and the IsRNA2+-predicted structure (c), but disrupted in the IsRNA2-predicted structure (b). From the secondary structure (d), it is clear that these residues are located at the ends of helices, therefore important for the determination of junction geometry.

The fourth example is one of the “difficult” cases: 3NPN; see Figure 8. In the structure predicted by IsRNA2 (Figure 8b), the loop containing residues 12–15, which are colored green, is attracted toward the middle region, leading to disruption of the stacking interactions. The stacking potential helps to restore the stacking interactions in the loop and corrects the orientation of the loop.

**Figure 8:**
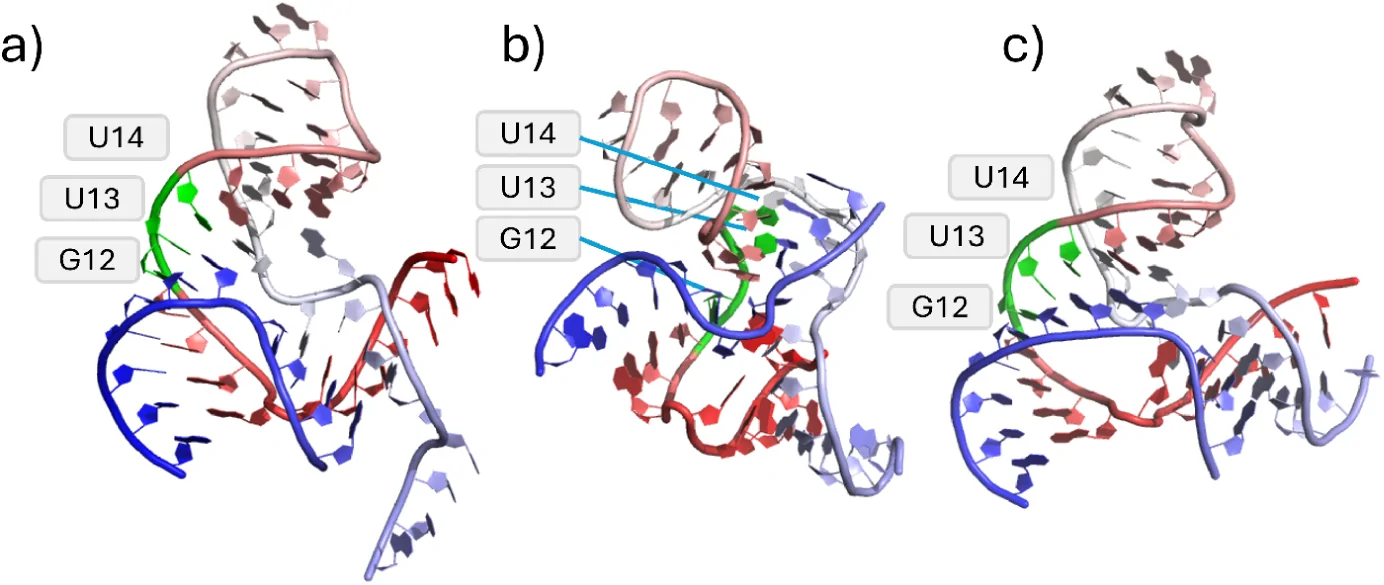
(a) Native structure; (b) structure predicted by IsRNA2; (c) structure predicted by IsRNA2+ of RNA 3NPN, respectively. Residues 12–15 are colored green and labeled. The three residues are stacked in both the native structure (a) and the IsRNA2+-predicted structure (c), but disrupted in the IsRNA2-predicted structure (b).

A recurrent theme is identified in the four examples described above. The attractive pairwise potential in IsRNA2 tends to bring different parts of the RNA molecule together, often leading to crowded regions in predicted structures. To assess the overall steric crowdedness of the structures, we calculated the radius of gyrations of the predicted and native structures of the four RNA molecules, presented in Table 5. Clearly, the IsRNA2-predicted structures have the smallest radius of gyration for all four cases. In order to avoid steric clashes in crowded regions, many stacked residue pairs in the loops are disrupted. The introduction of the explicit stacking potential counters the attractive force and restores the configurations of the loop regions, leading to more accurately predicted structures.

**Table 5:** Radius of Gyration (in Å) of the Native and Predicted Structures

	native	IsRNA2	IsRNA2+
1E95	14.57	13.53	14.10
2MTJ	18.83	16.90	17.06
8VM8	24.42	23.29	24.65
3NPN	18.09	15.57	17.32

Our results show that local interactions can play important parts in determining the overall tertiary structure of RNA molecules. The stacking potentials are trained on simple systems and applied only to adjacent bases in loops, but can lead to vast conformational changes in the predicted structures compared to the original IsRNA2 force field. This phenomenon stems from the fact that the overall structures of RNA molecules are often the combined results of a variety of long-range and local interactions, and a balanced representation of the important energy components is essential for accurate RNA force fields.

## Conclusion and Outlook

In this work, we present the iterative reweighting algorithm as a flexible and general method for optimizing force field parameters. We applied the procedure to train an explicit stacking potential that was augmented to the IsRNA2 force field. Explicit stacking terms are added to adjacent residues in loops. We name the modified force field the IsRNA2+ force field and applied it to structure prediction tasks on 80 systems with different complexities. The modified force field improved the accuracies of predicted structures in many systems, leading to about a 50% reduction in the predicted RMSD for some systems. We reason that the origin of this improvement lies in competition between the attractive pairwise potential and the stacking potential. Without the latter, distant parts of an RNA molecule often collapse together, resulting in distorted loops and disrupted base stacking. The explicit stacking potential helps to restore the correct orientations of loops, thus giving better-predicted structures.

We want to mention again that native secondary structure constraints are applied in benchmarking the structure-prediction performance. Apart from stabilizing the geometries of helical regions, the IsRNA2+ force field also relies on the secondary structure input to identify the loop regions. As a result, as is common for many tertiary structure prediction models, the performance of the IsRNA2+ force field depends on the reliability of the secondary structure input.

Another issue worth mentioning is the dependence of RNA base stacking on ionic conditions, as shown through experimental measurements.^66^ The IsRNA2+ stacking potential was optimized using reference MD trajectories generated at the standard physiological environment of 150 mM NaCl. Because of the implicit treatment of ions in the IsRNA2 force field, we are not able to test the transferability of this stacking potential to different ionic concentrations. In the meantime, various schemes exist for incorporating explicit ionic effects into coarse-grained force fields,^67–69^ and as the iterative reweighting method is a very general method for force field optimization, we propose that the current framework can be readily extended to force fields with explicit ions.

We also note that stacking interactions are ubiquitous in RNA molecules and do not limit to adjacent bases in loops. For example, in the L2 loop of RNA 1E95 (Figure 5d), one residue protrudes out from the loop, and the two residues before and after it are stacked with each other. In other cases, bases that are even farther apart can be stacked. Such stacking interactions are often important for the formation of tertiary motifs and thus crucial to the determination of the overall tertiary structures. RNA 1RMN (Figure 9) among the test cases is another example. Residues 9,10 and 27,28 reside on two different loops in the junction region, but they are intercalated and stacked with each other. This stacked segment is important for determining the orientations of the helices around the junction.

**Figure 9:**
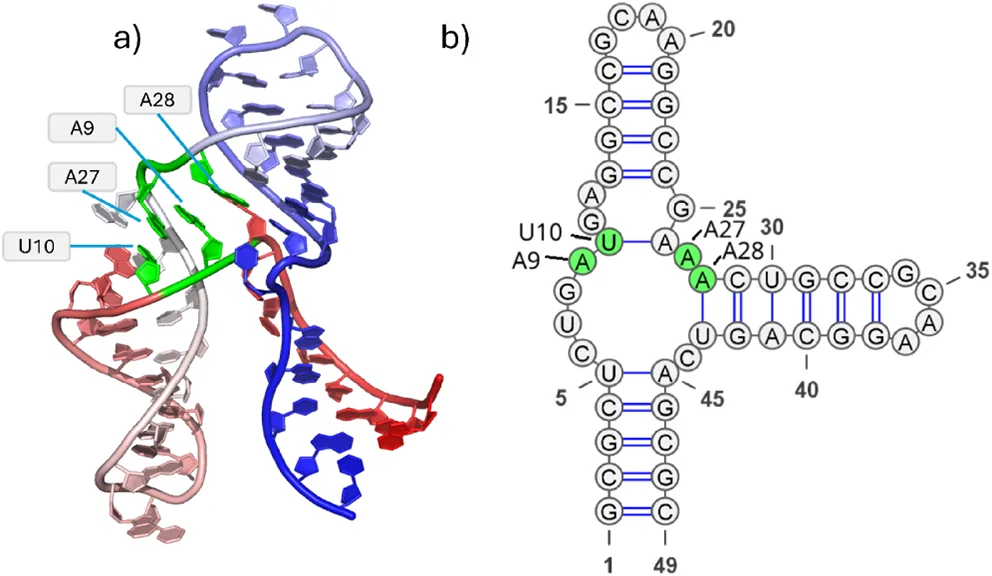
Native and 2D structures of RNA 1RMN. Residues 9, 10, 27, and 28 are colored green and labeled. The four residues are from two separate segments of the RNA molecule and stacked in an alternating pattern.

We performed a more comprehensive analysis across the test cases by structure analysis using the x3DNA-DSSR package,^62^ and the results show that 42 out of 46 simple systems and all 20 difficult systems have nonlocal stacked pairs. The existence of extensive nonlocal stacking is often associated with larger, more complicated systems that are challenging for structure prediction. For example, in the simple systems, there are 4 simple systems where RMSD of the IsRNA2+ predicted structure exceeds 10 Å: 1U9S, 2MHI, 2NBX, and 9IWF, with sequence lengths of 155, 53, 108, and 69, respectively. Of these systems, 1U9S contains an exceptionally large number of 28 nonlocal stacked pairs, while the other 3 systems also contain multiple nonlocal stacked pairs.

Therefore, it is conceivable that applying stacking potentials to both local and nonlocal pairs of residues would also be important in structure prediction. We carried out further exploratory tests by applying the stacking potentials additionally to all native nonlocal stacked pairs. We would like to note that this extrapolation can only be viewed as a proof of concept to examine if including nonlocal stacked pairs can further improve the accuracy of structure prediction. This procedure will not be practical in an actual structure prediction task as we rely explicitly on the prior knowledge of the native nonlocal stacked pairs. This information will be absent without knowing the native structure, which then defeats the purpose of structure prediction. Furthermore, the stacking potentials were not trained on these nonlocal stacked bases, and it is thus not strictly rigorous to extend them to nonlocal stacked pairs.

We selected 15 simple systems with multiple nonlocal stacked pairs and ran structure prediction with nonlocal stacked pairs using the same procedure. Our selection primarily targeted systems where IsRNA2+ with local stacking gave large RMSDs and possessed multiple nonlocal stacked pairs. Specifically, the IsRNA2+-predicted structures of the selected 15 systems had an average RMSD of 8.60 Å and an average of 6.7 nonlocal stacked pairs, much higher than the rest of the systems (on average 5.45 Å and 3.7 pairs, respectively). A scatterplot showing the distribution of IsRNA2+ results and the number of nonlocal stacked pairs of the systems can be found in Supporting Information Figure S9. The structure prediction results are summarized in Table 6. In 10 out of the 15 cases, the additional stacking terms led to further improvements in the predicted structures, reducing the overall average RMSD of the selected set from 8.60 Å to 6.83 Å. Notably, the RMSDs of 1U9S and 9IWF both more than halved compared to predictions with IsRNA2+ where stacking terms were applied only to adjacent residues.

**Table 6:** RMSD (in Å) of the Predicted Structures of 15 Simple Systems with Nonlocal Stacked Pairs Using IsRNA2 and IsRNA2+ Force Fields, Measured on Heavy Atoms^a^

	IsRNA2	IsRNA2+	IsRNA2+ (nonlocal pairs)
1U9S	15.30	15.06	**6.42**
2LU0	**7.28**	9.58	7.36
2MHI	14.80	12.04	**9.45**
2MTJ	10.56	**4.17**	7.95
2NBX	14.60	15.79	**11.26**
2OIU	12.10	9.37	**8.94**
2P89	7.71	**6.77**	7.81
2QUS	8.20	5.39	**4.47**
3E5C	7.71	7.68	**5.46**
3LA5	9.80	**5.33**	6.42
3ZD5	7.95	6.89	**5.26**
4KZ2	**5.17**	5.59	5.87
4LVW	**3.73**	3.79	5.18
8VM8	15.33	8.97	**5.20**
9IWF	11.57	12.52	**5.42**
**Average**	10.12	8.60	**6.83**

a For each RNA molecule, the best result is shown in bold.

In the meantime, to ensure a balanced evaluation, we also included several control systems where IsRNA2+ with only adjacent pairs already predicted structures with RMSD lower or close to 5 Å, such as 2MTJ, 2QUS, 3LA5, 4KZ2 and 4LVW. The cases are included to test if the additional nonlocal stacking interactions might lead to disruptions of the predicted structures. And such deterioration was indeed observed, notably in 2MTJ, where RMSD went up from 4.17 Å to 7.95 Å. It is therefore conceivable that such deterioration may occur for other untested systems where the IsRNA2+ predicted structures already produced low RMSD. One possible factor contributing to the degradation of performance is that the applied stacking potential was trained on adjacent stacked pairs only and should not be extended to nonlocal cases. Therefore, we would like to note again that this test is mostly intended as a proof of concept and points to possible directions for future exploration.

For the current work, we chose to restrict ourselves to adjacent residues in loops, and two challenges must be resolved in the development of a stacking potential for nonlocal pairs. First of all, unlike in the case of adjacent residues in loops, stacking with other residues involves many different types. In cases like 1E95, stacking is on next-adjacent residues in a loop; in cases like 1RMN, stacking is between residues on different loops in the same junction region. There are other cases, for example, where bases on distant loops may insert into a helical region and stack with the residues in the helix. It is expected that different types of stacking interactions show different geometrical preferences, and a systematic characterization and parametrization would be challenging. More specifically, a recent structural survey^49^ showed that the stacking geometry between consecutive bases is structurally constrained to cis-orientations, leading to a narrow distribution of the glycosidic angles with a single peak at around 30^◦^. In contrast, nonconsecutive (nonlocal) stacked pairs display higher structural variability, showing both cis- and trans-orientations. Although our potential is able to distinguish between cis- and trans-configurations, it will be challenging to construct a reference training set to ensure balanced representations of the two cases, and the well-defined distribution of the consecutive bases provides a more robust baseline to test the performance of the iterative reweighting training algorithm. And as our stacking potential is a statistical potential, its application is most rigorous when restricted to the structural context of its training. So we only apply it to adjacent residues in loops, unlike HiRE-RNA^14^ where the analytical stacking term can be applied to all pairs.

On the other hand, we also note that the oxRNA^64^ force field contains different stacking terms for different scenarios: *V*_*stack*_ between adjacent bases on a strand, *V*_*cr.st.*_ across the duplex axis, and *V*_*coax*_ for coaxial stacking. A future direction of exploration would be to follow the practice and train separate potentials for both the adjacent and the cross types.

Second, in tertiary structure prediction tasks, it is often the case that secondary structures are available either from experimental measurements or from secondary structure predictions. In such cases, a stacking potential can be applied to residues belonging to loops. But for nonlocal pairs of residues, there is no such prior knowledge that could allow us to know which pairs would be stacked. Applying stacking potentials to all possible residue pairs can be computationally prohibitive, especially for larger systems. On the other hand, if assumptions can be made about the approximate spatial arrangements of different parts of the RNA molecules, then a stacking potential may be added to all residue pairs belonging to segments that are close to each other. However, caution should be taken to ensure that such assumptions are reliable to avoid the introduction of non-native interactions.

The two difficulties described above may be partially alleviated by taking into account the rapid development of machine learning methods in the field of RNA structure prediction. Random forest models may be useful in organizing different types of stacking interactions, and models may also be developed to predict possible stacked residues based on sequence and secondary structure inputs. Another possibility is to incorporate the results of end-to-end structure prediction models like AlphaFold3.^31^ As discussed before, while the latter models have achieved remarkable success in structure prediction, they suffer from the limitation of insufficient training data and do not provide the dynamical information required for understanding processes such as the folding of RNA molecules. Adopting tertiary structure information from machine learning models as constraints in physics-based force fields offers a promising middle ground that combines the strengths of both worlds.^70^

Furthermore, the iterative reweighting procedure demonstrated in the current work is a general framework for the optimization of force field parameters. While this work focused on the explicit stacking potential, it can also be applied to add or refine other force field terms, such as tertiary interactions or ligand-binding potentials. Since correlations between different degrees of freedom are contained in the training process, we propose that the iterative reweighting procedure will offer a useful and flexible choice in the toolbox of force-field development, especially for the optimization of complicated many-body potentials.

## Supplementary Material



## Data Availability

The data underlying this study are available in the published article and the Supporting Information, including the tabulated distance and angle potentials.

## Notes

The tabulated distance and angle potentials for purine–purine, purine–pyrimidine, and pyrimidine–pyrimidine stacking; histograms of loop lengths and purine fractions of the test set; various figures showing the trained potentials as well as training curves; tables containing oxRNA and HiRE-RNA results (PDF)
