# Supplementary material for: Developing Explicit Base Stacking Potentials for the IsRNA2+ Coarse-Grained RNA Force Field Using Iterative Reweighting
Source: J Chem Theory Comput. 2026 Sep 2;22(18):9762–75. doi: 10.1021/acs.jctc.6c01116 (PMC13602889; doi:10.1021/acs.jctc.6c01116)
Supplement: Supplementary file 1 [file ct6c01116_si_001.pdf]

Supplementary Information

Wenfei Li[1] and Shi-Jie Chen[1,2,*]

[1] Department of Physics and Astronomy, University of Missouri-Columbia, Columbia, MO 65211-7010, USA

[2] Department of Biochemistry, MU Institute of Data Science and Informatics, University of Missouri-Columbia, Columbia, MO 65211-7010, USA

[*]To whom correspondence should be addressed. E-mail: chenshi@missouri.edu

Supplementary Figures

S1 Histogram of loop lengths by loop classes and system types in the testing set. Vertical lines indicate range of loop lengths in the training set, with fractions of systems falling in the range indicated in the figure. 3
S2 Histogram of purine fractions by loop classes and system types in the testing set. Vertical lines indicate range of purine fractions in the training set, with fractions of systems falling in the range indicated in the figure. 4
S3 Tradeoff between Jensen-Shannon divergence and smoothness loss with different λ. . 4
S4 Trained potentials with different λ. 5
S5 Loss curves across 3 iterations of the training process. 5
S6 Loss curves with different learning rates. 6
S7 Trained potentials with different learning rates. 6
S8 Trained potentials as well as observed distributions from CGMD simulations across different iterations. 7
S9 Scatterplot of IsRNA2+ results against number of non-local stacking pairs for selected simple systems where predictions were carried out with additional non-local stacking, with the 4 systems with largest predicted RMSDs labeled. 8

Supplementary Tables

S1 RMSD (in Å) of the predicted structures of 14 systems in the training set using different methods, measured on heavy atoms. 9
S2 RMSD (in Å) of the predicted structures of 60 simpler systems using oxRNA and HiRE-RNA, including both the 46 systems independent from the training set, as well as the 14 systems in the training set, with the respective averages. 10
S3 RMSD (in Å) of the predicted structures of 20 difficult systems using oxRNA, measured on heavy atoms. 11

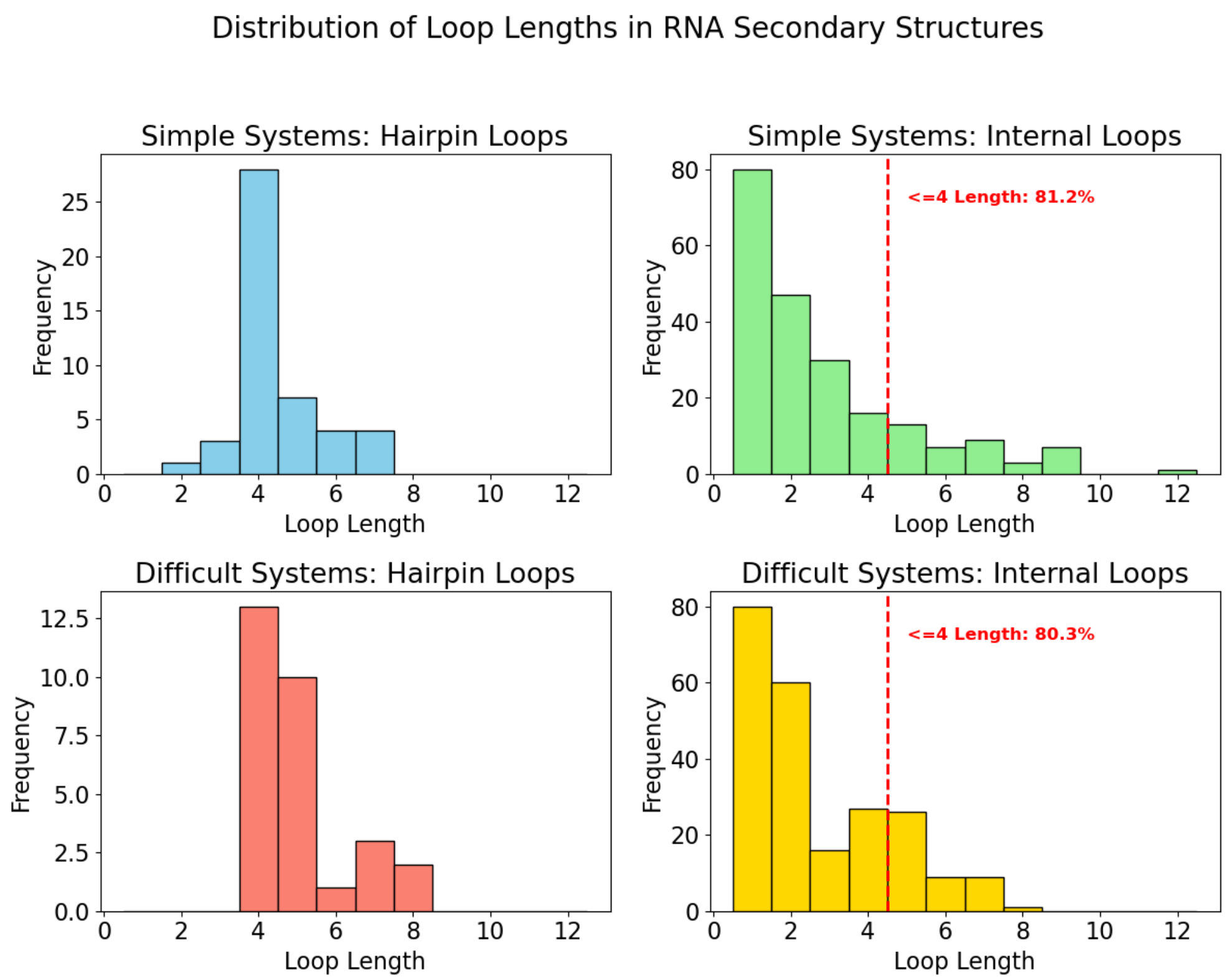

Figure S1: Histogram of loop lengths by loop classes and system types in the testing set. Vertical lines indicate range of loop lengths in the training set, with fractions of systems falling in the range indicated in the figure.

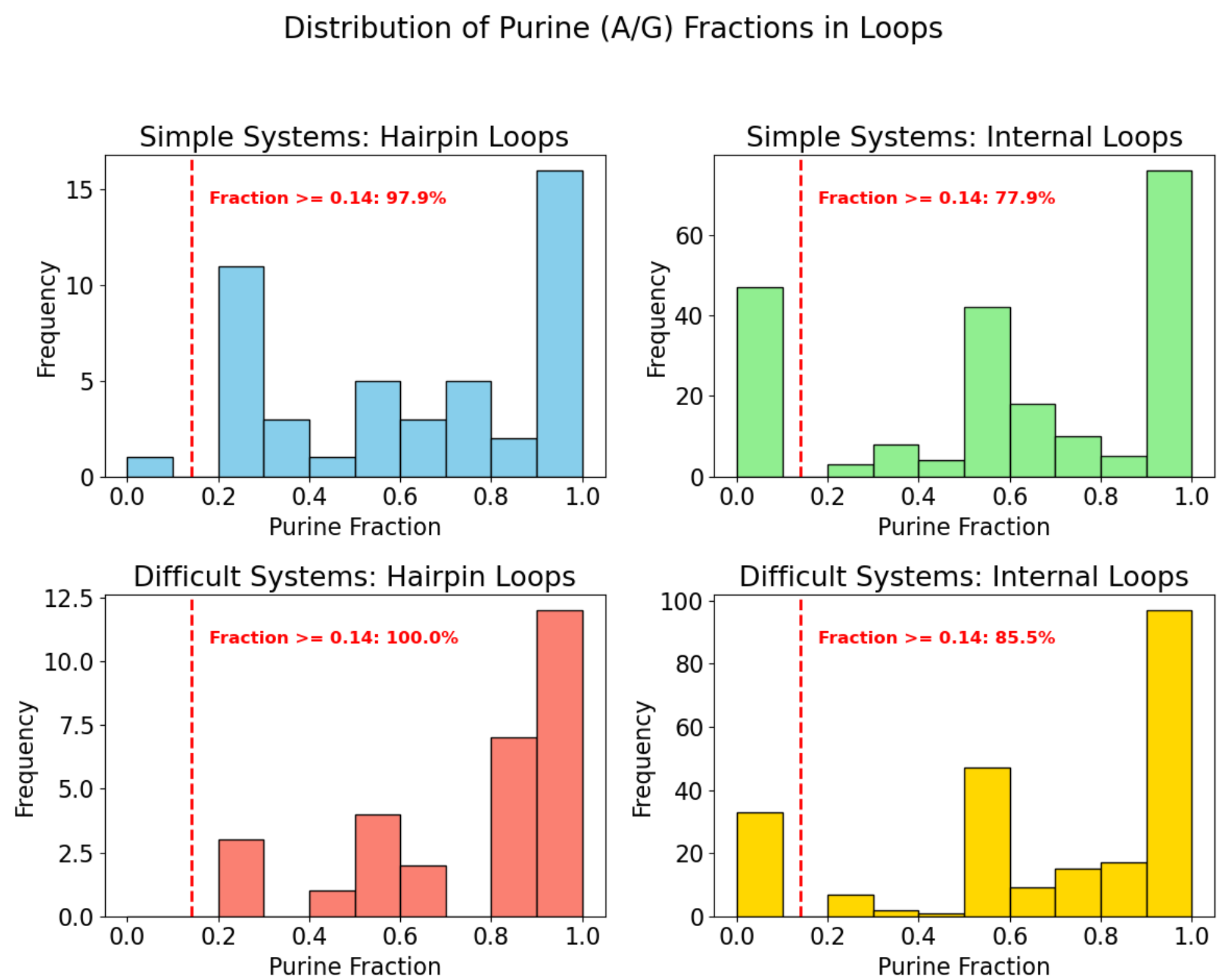

Figure S2: Histogram of purine fractions by loop classes and system types in the testing set. Vertical lines indicate range of purine fractions in the training set, with fractions of systems falling in the range indicated in the figure.

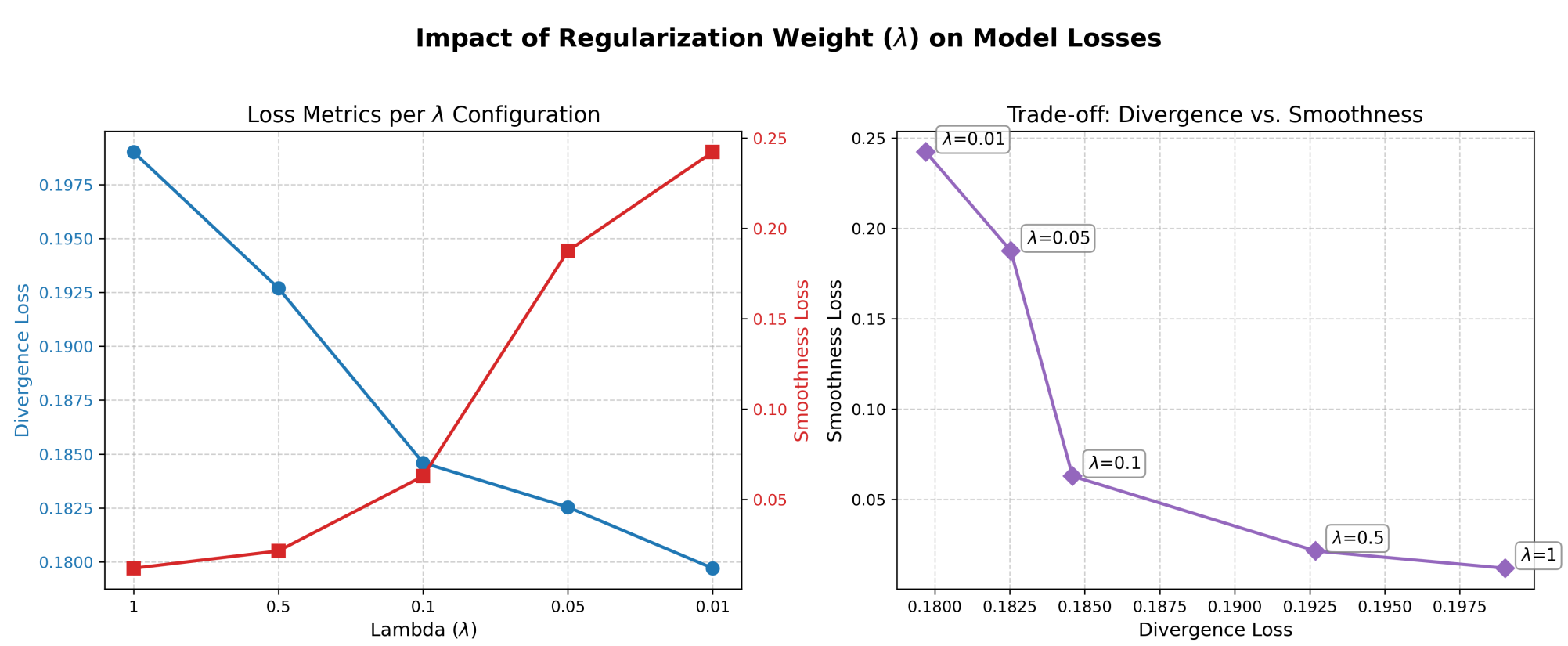

Figure S3: Tradeoff between Jensen-Shannon divergence and smoothness loss with different λ.

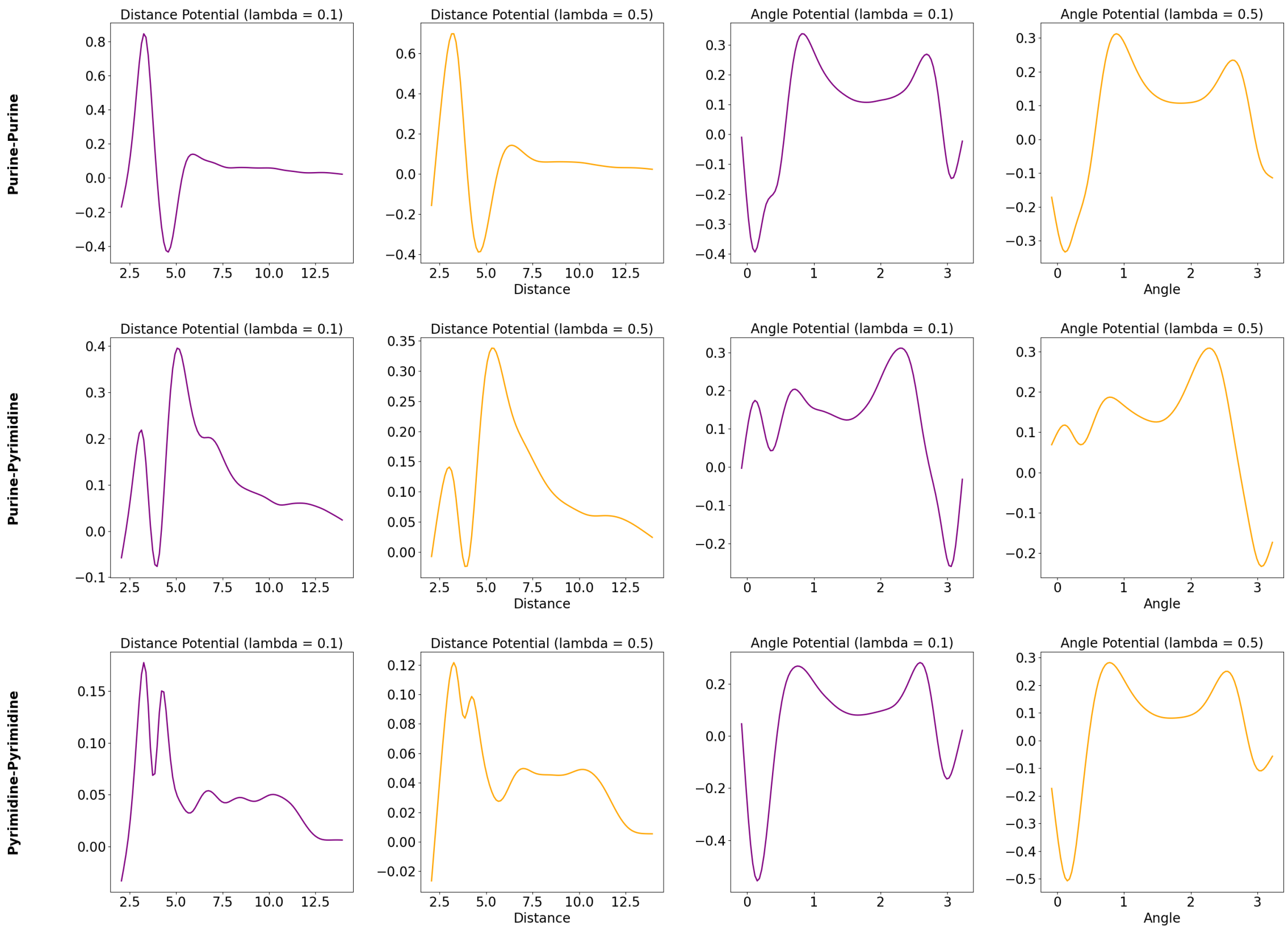

Figure S4: Trained potentials with different λ.

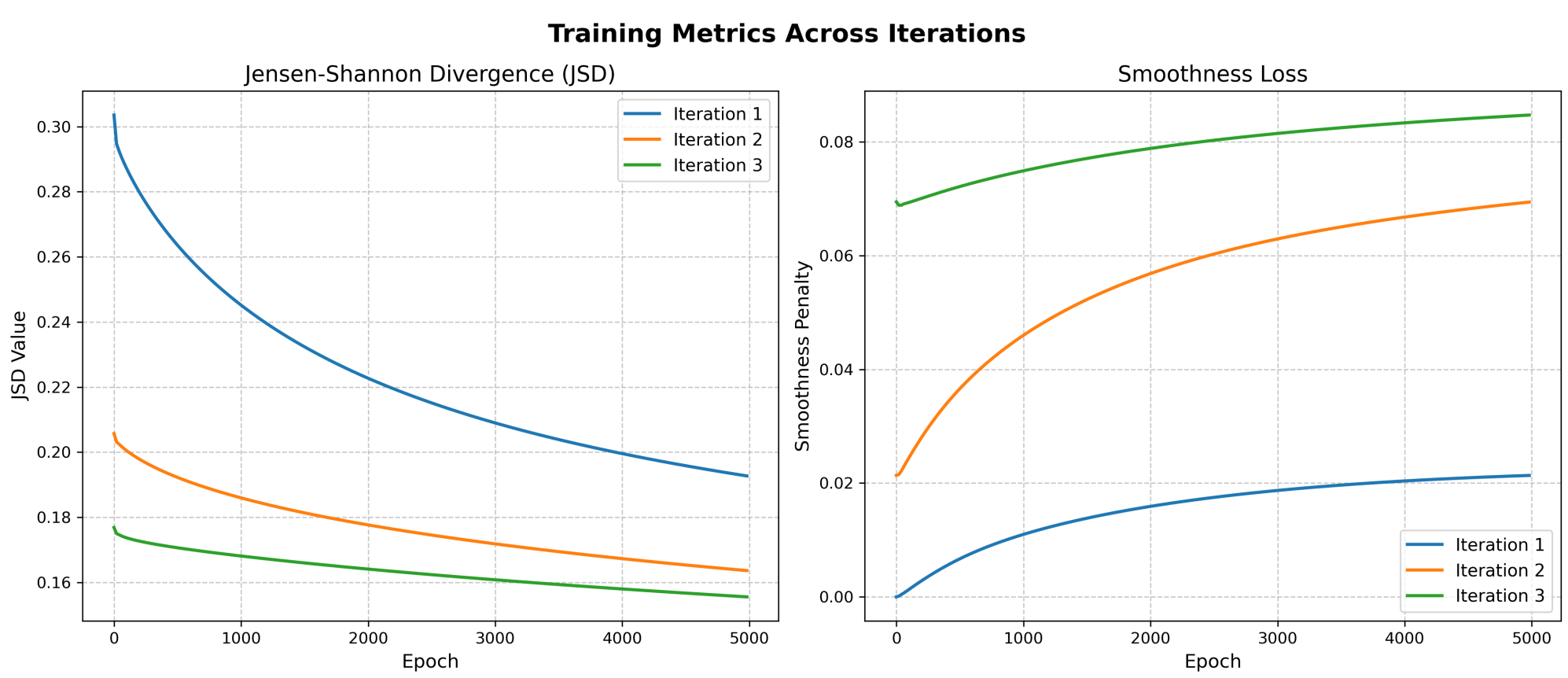

Figure S5: Loss curves across 3 iterations of the training process.

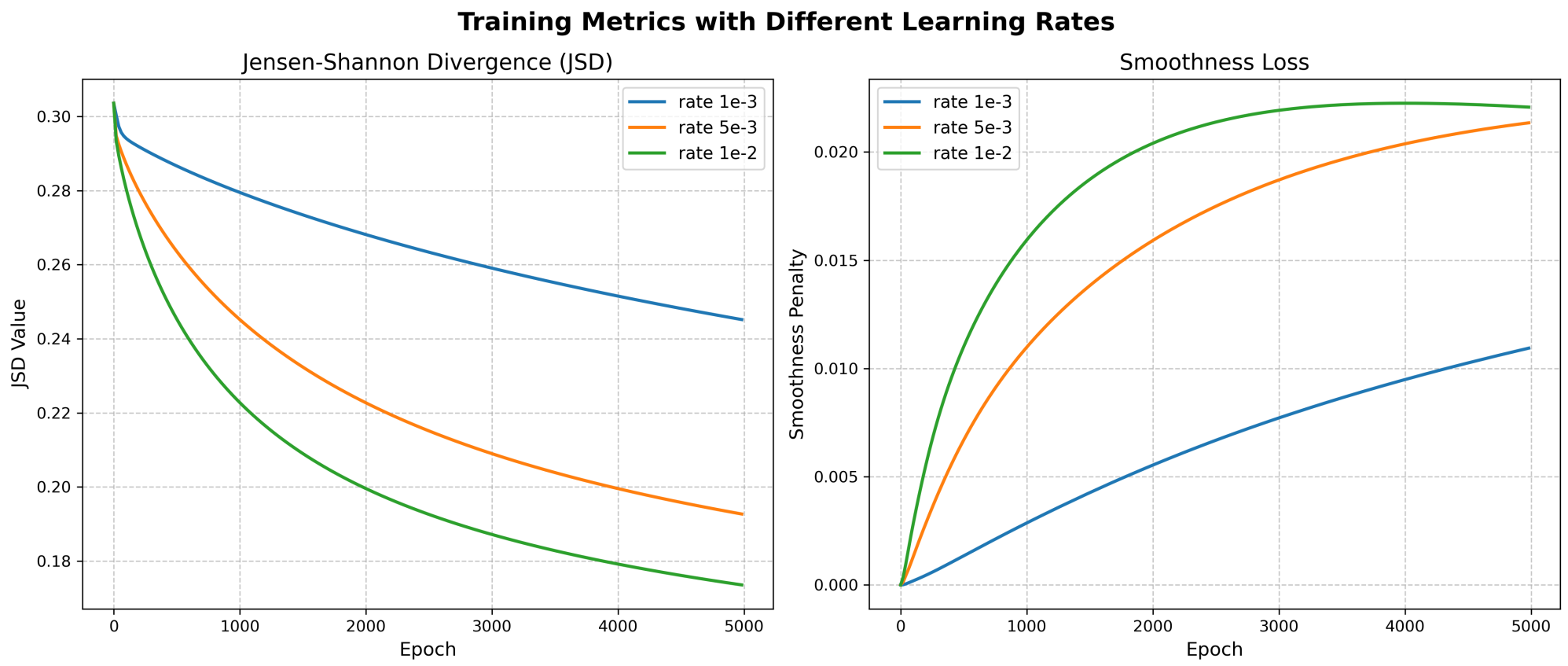

Figure S6: Loss curves with different learning rates.

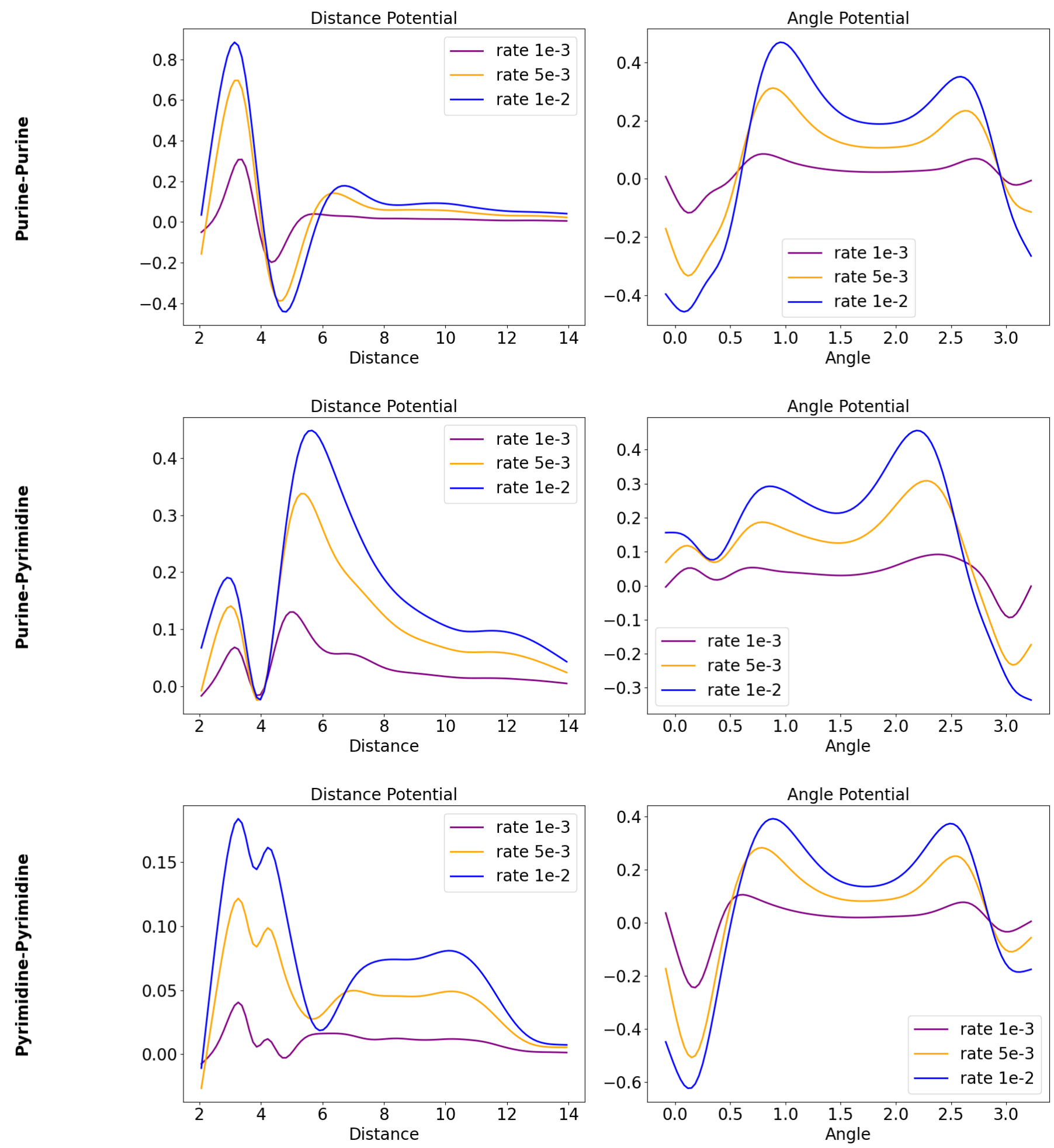

Figure S7: Trained potentials with different learning rates.

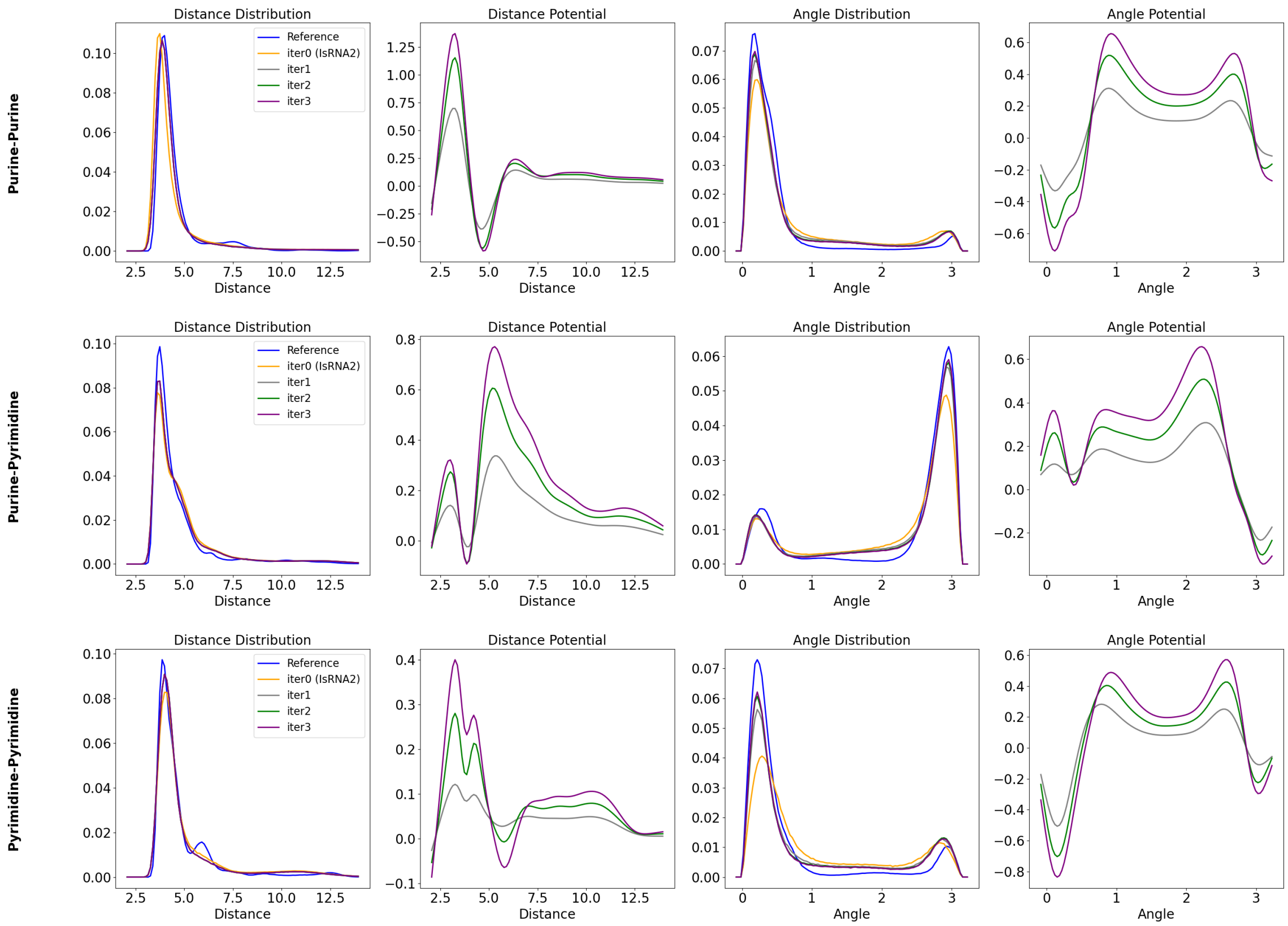

Figure S8: Trained potentials as well as observed distributions from CGMD simulations across different iterations.

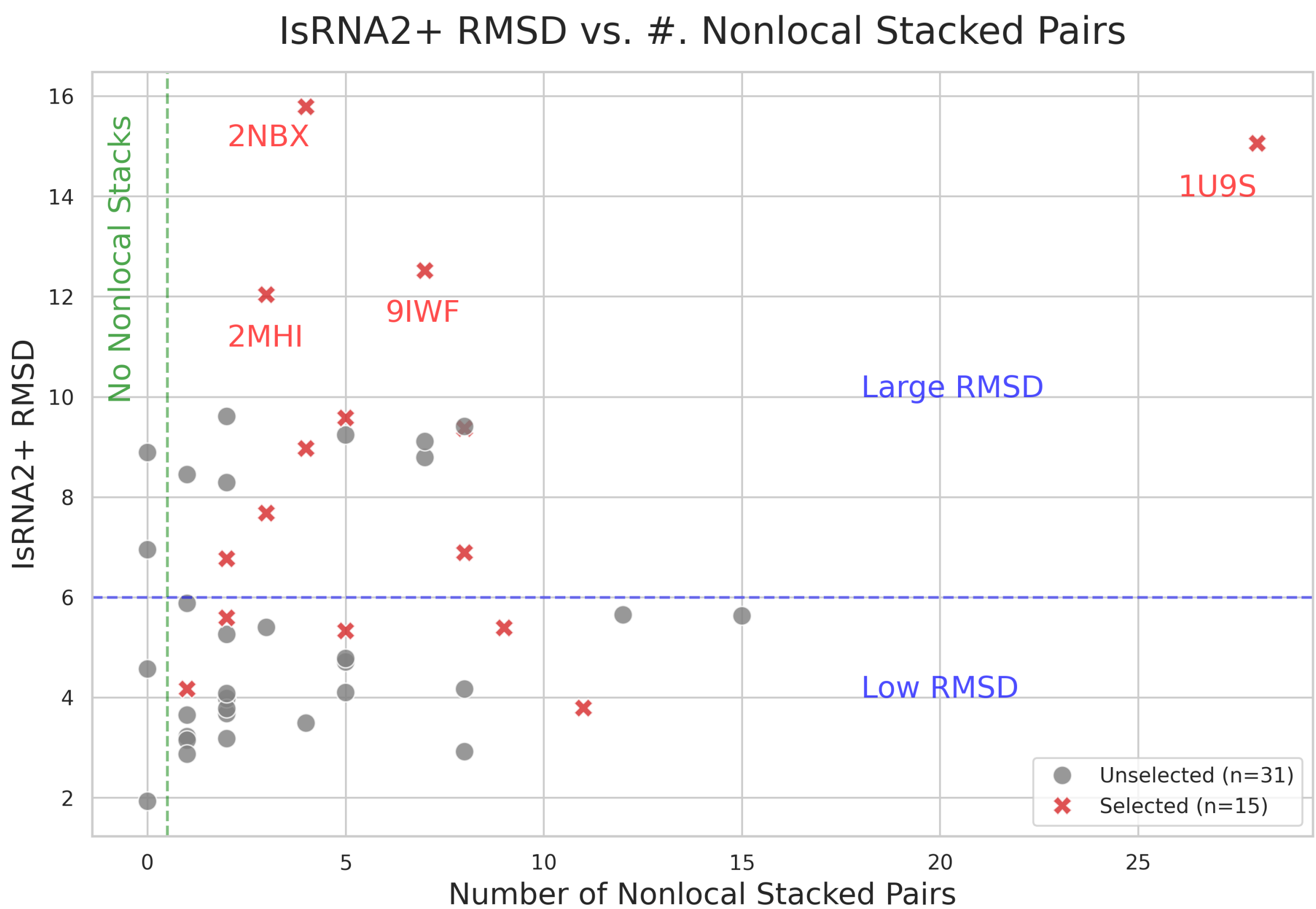

Figure S9: Scatterplot of IsRNA2+ results against number of non-local stacking pairs for selected simple systems where predictions were carried out with additional non-local stacking, with the 4 systems with largest predicted RMSDs labeled.

	IsRNA2	IsRNA2+	SimRNA	iFoldRNA	FARFAR2	AlphaFold3	RNAJP
157D	1.24	**0.97**	1.56	-	2.96	0.68	1.08
1ATO	3.66	**3.18**	3.29	3.63	4.15	2.58	4.43
1D0U	3.02	**2.13**	2.50	3.04	2.76	1.32	3.15
1F85	2.53	**1.66**	1.98	2.88	2.99	0.95	2.77
1F9L	2.56	1.98	2.01	2.19	3.80	1.58	**1.83**
1FHK	2.12	1.87	2.00	2.99	3.66	1.05	**1.61**
1K5I	1.72	1.65	**1.37**	2.07	2.59	1.20	2.09
1KKA	**2.91**	2.97	3.54	4.51	4.95	2.97	5.09
1T28	4.24	4.32	**4.08**	4.77	8.68	3.46	7.31
2L8F	**2.69**	2.80	4.18	-	3.06	0.78	6.54
2LDL	3.16	**2.67**	2.95	4.33	3.70	2.06	3.16
2LX1	6.52	5.90	5.56	-	6.98	1.18	**5.46**
2NBY	3.35	**2.72**	3.29	3.94	4.28	1.45	6.38
402D	0.97	**0.86**	1.47	-	2.96	0.72	1.56
**Average**	2.91	2.55	2.84	3.44	4.11	1.57	3.75

Table S1: RMSD (in Å) of the predicted structures of 14 systems in the training set using different methods, measured on heavy atoms.

	oxRNA*	HiRE-RNA†
**Independent Test Set (46 Unseen Systems)**		
1A60	15.75	-
1E95	5.31	9.52
1FQZ	5.21	6.79
1KAJ	9.79	13.60
1KP7	5.98	6.54
1KPD	7.56	7.96
1L2X	4.24	9.16
1P5N	4.39	5.06
1Q9A	4.55	6.72
1RMN	11.09	-
1RNK	5.99	20.50
1U9S	-	-
1YG4	11.35	10.65
1YKQ	11.20	-
1YSV	2.82	3.69
299D	10.33	-
2A43	6.67	17.37
2AP5	11.20	9.31
2CKY	19.26	-
2G1W	10.01	14.88
2LDZ	4.09	3.84
2LU0	10.93	-
2MHI	13.45	-

2MTJ	10.20	-
2N6Q	3.49	6.86
2N8V	17.34	-
2NBX	11.39	-
2NC1	16.72	-
2OIU	12.18	-
2P89	7.29	-
2QUS	16.59	-
2QWY	16.11	-
2RP1	7.30	6.72
2TPK	5.40	8.54
3E5C	18.71	-
3LA5	10.71	-
3VRS	9.48	-
3ZD5	14.89	-
4JF2	9.98	-
4KZ2	12.50	-
4LVW	15.23	-
4OJI	11.02	-
4RGE	10.24	-
5KH8	9.39	-
8VM8	15.80	-
9IWF	13.39	-
**Average**	10.37	9.32
**Training Set Systems (14 Systems)**		
157D	1.79	8.26
1ATO	3.67	3.34
1D0U	3.00	6.38
1F85	2.73	3.95
1F9L	3.43	6.21
1FHK	1.98	6.46
1K5I	2.80	2.83
1KKA	4.18	3.83
1T28	4.64	10.28
2L8F	2.99	9.52
2LDL	3.63	4.63
2LX1	6.06	5.60
2NBY	3.77	-
402D	11.38	2.70
**Average**	4.00	5.69

Table S2: RMSD (in Å) of the predicted structures of 60 simpler systems using oxRNA and HiRE-RNA, including both the 46 systems independent from the training set, as well as the 14 systems in the training set, with the respective averages.

Note: We would like to mention that both OxRNA and HiRE-RNA are not specifically designed for RNA

structure prediction task. OxRNA is intended for exploring structural, mechanical, thermodynamics of RNA molecules[1] and can be applied to very large systems. Hire-RNA has been applied extensively to study RNA folding dynamics[2, 3]. *For oxRNA structures, RMSDs are measured on heavy atoms. For several systems, the clustering procedure failed to produce meaningful clusters from oxRNA trajectories, and the results are indicated by '-'. †We were unable to find relevant tools to convert HiRE-RNA structure files back to all-atom ones, so the RMSDs were evaluated based on the coarse-grained HiRE-RNA representations. Also, in order to model folding dynamics, HiRE-RNA does not use secondary structure constraints, and in most cases we observed unraveling of RNA helices during the REMD simulations. HiRE-RNA predictions were restricted to 30 systems in the 'simple' test set with fewer than 40 residues to ensure computational feasibility, and the missing values are indicated by -.

	oxRNA
2G9C	5.30
2XO1	10.79
3D0U	-
3DIG	-
3DS7	12.47
3F2Q	20.97
3GX2	16.17
3NPN	11.86
3RKF	14.22
3SKL	13.19
4GXY	-
4L81	13.43
4QK8	19.81
4QLM	14.71
4Y1M	11.23
5KPY	13.34
6N5K	20.47
6N5T	21.74
6VMY	-
7KVT	-
**Average**	14.65

Table S3: RMSD (in Å) of the predicted structures of 20 difficult systems using oxRNA, measured on heavy atoms.

Note: See note of Table S2.

References

[1] Petr Šulc, Flavio Romano, Thomas E Ouldridge, Jonathan PK Doye, and Ard A Louis. A nucleotide-level coarse-grained model of rna. *The Journal of chemical physics*, 140(23), 2014.

[2] Tristan Cragnolini, Yoann Laurin, Philippe Derreumaux, and Samuela Pasquali. Coarse-grained hire-rna model for ab initio rna folding beyond simple molecules, including noncanonical and multiple base pairings. *Journal of chemical theory and computation*, 11(7):3510–3522, 2015.

[3] Samuela Pasquali and Philippe Derreumaux. Hire-rna: a high resolution coarse-grained energy model for rna. *The journal of physical chemistry B*, 114(37):11957–11966, 2010.
